# Bioremediation of Produced Water by a Polyextremophilic, Heavy-Metal-Resistant *Modicisalibacter* sp. Strain Wilcox

**DOI:** 10.1021/acsestwater.5c01373

**Published:** 2026-03-26

**Authors:** Damilare Ajagbe, Mark Krzmarzick, Babu Fathepure

**Affiliations:** † Department of Microbiology and Molecular Genetics, 7618 Oklahoma State University, Stillwater, Oklahoma 74078, United States; ‡ Department of Civil and Environmental Engineering, Oklahoma State University, Stillwater, Oklahoma 74078, United States

**Keywords:** produced water, bioremediation, heavy metals, polyextremophile, salinity, hydrocarbons, biosorption, biorecovery

## Abstract

Produced water (PW), generated during oil and gas extraction, is a complex wastewater characterized by high salinity, hydrocarbons, and heavy metals, making treatment and beneficial reuse challenging. Bioremediation offers a sustainable treatment alternative, but the extreme physicochemical conditions of PW inhibit the growth and activity of most conventional microorganisms. This study evaluates the bioremediation potential and heavy metal tolerance mechanisms of the halophile, *Modicisalibacter* sp. strain Wilcox, isolated from PW. We examined its growth and benzene, toluene, ethylbenzene, and xylenes (BTEX) degradation capability under elevated salinity in defined media and raw PW, while assessing the effects and fate of metals individually and in multimetal mixtures. Strain Wilcox demonstrated exceptional tolerance to individual metals, including 100 mM arsenate, 100 mM manganese, 12.5 mM cadmium, and 7 mM zinc. Increasing metal concentrations and multimetal mixtures reduced BTEX degradation rates, with toxicity varying by metal species and salinity. In addition to hydrocarbon degradation, the strain removed 75−99% of Mn^2+^, Zn^2+^, Se^4+^, Pb^2+^, Cr^3+^, and Cu^2+^ via biosorption and bioaccumulation. Functional genomic analysis supported these phenotypes, revealing >70 metal resistance genes, 58 osmoregulation genes, and ∼70 genes associated with cross-protection against salt and metal stress, highlighting strain Wilcox’s potential for bioremediation of PW.

## Graphical abstract

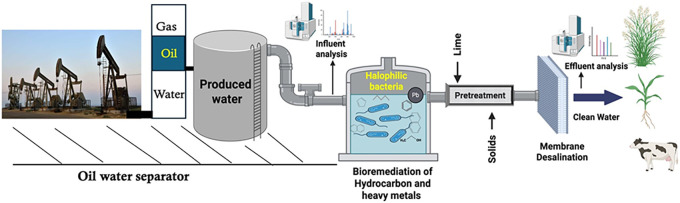

## Introduction

Extremophiles, microorganisms that thrive in harsh environmental conditions, are of significant interest for their potential in biotechnology and bioremediation. These unique organisms can survive in habitats characterized by extremes in temperature, pH, salinity, pressure, and the presence of toxic compounds.^1^ Among them, halophiles, which thrive in high-salinity environments, play a crucial role in the degradation of pollutants including hydrocarbons, pesticides, heavy metals, and other organic contaminants in saline or hypersaline environments where conventional microbes cannot survive.^2–6^

Produced water (PW), a byproduct of oil and natural gas production, presents a considerable challenge for remediation due to its polyextreme nature. Each year, approximately 25 billion barrels of PW are produced in the United States and over 90 billion barrels globally.^7–9^ Growing concerns over water scarcity, intensified by climate change and increasing demand from both the public and industry, have renewed interest in treating PW as an alternative water resource. Transforming this abundant but complex waste stream into a reliable source of clean water could help mitigate environmental impacts while contributing to more sustainable water supplies.

PW is chemically complex, containing extreme levels of salt (up to 32% NaCl), petroleum hydrocarbons, organic acids, fracking additives, naturally occurring radioactive materials (NORM), and heavy metals.^8–11^ Conventional treatment methods are often ineffective, while advanced electrochemical and thermal technologies, although promising, are typically cost- and energy-intensive. While bioremediation is considered a potential alternative, the combined stressors make PW a polyextreme environment that severely limits microbial survival, thereby reducing the effectivity of most bioremediation strategies. Successful bioremediation of PW therefore requires specialized microorganisms capable of degrading hydrocarbons at high salinity while also tolerating and detoxifying heavy metals.

Heavy metals are of particular concern due to their persistence, nondegradable nature, and potential to accumulate in the environment.^12–14^ While trace metals such as Cu, Zn, Mn, Ni, Co, and Fe serve as essential micronutrients, they become toxic at elevated concentrations. Others, including Al, Hg, As, Cd, and Pb, have no known biological function and are toxic at even low concentrations.^15,16^ These metals exert their toxicity through multiple mechanisms, including oxidative stress, disruption of protein and membrane function, interference with essential transport processes, and DNA damage.^17–19^ Therefore, understanding how microorganisms cope with such a blend of stressors is crucial for predicting microbial activity in these environments and formulating effective bioremediation strategies in complex extreme environments like PW. Previous studies have mostly focused on the effect of individual stressors such as salinity or specific metals on growth and survival strategies by microbes, with few addressing their combined impact.^20–22^

This study investigates a halophilic bacterium, *Modicisalibacter* sp. strain Wilcox, isolated from PW from the First Wilcox formation in Oklahoma.^23^ Known for its ability to degrade various hydrocarbons under highly saline conditions, this bacterium serves as an ideal model for investigating microbial adaptation and survival in polyextreme environments. To mimic the complex conditions of PW, we systematically examined the bacterium’s ability to degrade benzene, toluene, ethylbenzene, and xylenes (BTEX) as the sole carbon sources in the presence of varying levels of salinity and heavy metals. We first assessed tolerance to individual heavy metals under high salinity and then evaluated combinations of metals, better reflecting real-world scenarios. We also investigated the impact of varying salt concentrations on both metal tolerance and hydrocarbon degradation and explored the bacterium’s metal removal capabilities via biosorption and bioaccumulation mechanisms. We further elucidated the molecular mechanisms underlying strain Wilcox’s metal resilience and survival using genomic analysis. Finally, we evaluated the bacterium’s growth and metal tolerance in raw PW.

Overall, this study offers new insights into microbial survival under extreme conditions and demonstrates the potential of strain Wilcox for the bioremediation of hydrocarbons and heavy metals in hypersaline wastewaters, such as PW.

## Materials and Methods

### Chemicals and Growth Medium

All chemicals used in this study were of analytical grade and were used as received without any further purification. Benzene, toluene, ethylbenzene, xylenes, copper (ii) chloride dihydrate (CuCl_2_.2H_2_O), nickel (ii) chloride hexahydrate (NiCl_2_·6H_2_0), cobalt (ii) chloride hexahydrate (CoCl_2_·6H_2_0), sodium selenite (iv) pentahydrate (Na_2_SeO_3_·5H_2_O), lead (ii) nitrate (Pb (NO_3_)_2_), manganese (ii) sulfate monohydrate (MnSO_4_·H_2_O), cadmium (ii) chloride (CdCl_2_), and chromium (iii) chloride hexahydrate (CrCl_3_·6H_2_0) were purchased from Sigma-Aldrich, USA. Sodium hydrogen arsenate heptahydrate (Na_2_HAsO_4_·7H_2_O) and Zinc chloride (ZnCl_2_) were procured from Thermo Fisher Scientific, USA.

### Mother Culture

Strain Wilcox was maintained in 1-L bottles with 500 mL of mineral salts medium (MSM) supplemented with 2.5 M NaCl (14.5% NaCl) and 30 μL of neat (250−350 μmole) of each BTEX compound as the sole source of carbon and energy.^23^ Bottles were closed with Teflon-coated septa and aluminum caps. Headspace was withdrawn periodically and monitored for consumption of the added BTEX. Roughly once every 4 weeks, 50% of the culture was replaced with fresh MSM containing 2.5 M NaCl and 250−350 μmole of each BTEX compound.^23^ This culture served as the source of the inoculum for all experiments presented in this article unless stated otherwise.

Unless otherwise stated, all experiments were carried out in triplicate using 160 mL capacity serum bottles containing 49 mL of MSM supplemented with 2.5 M NaCl (14.5% NaCl).^24^ The bottles were spiked with 3 μL of neat (25 − 35 μmole) of each BTEX compound. Individual metals or a mixture of metals were added to the bottles from individual stocks. Bottles were inoculated with 1 mL of strain Wilcox from the mother culture (approximately 5 × 10^5^ CFU/mL). Bottles were sealed with Teflon-coated septa and aluminum caps. Air in the headspace served as the source of oxygen. BTEX was monitored by regularly withdrawing headspace gas and injecting into a Hewlett-Packard 6890 gas chromatograph as previously described.^25^ Complete degradation of the BTEX compounds was defined by the disappearance of BTEX from the headspace as measured by a gas chromatograph and compared to the abiotic controls.

### Heavy Metal Tolerance and Tolerance Mechanisms in Strain Wilcox

This study evaluated the effects of various heavy metals, both individually and in combination (as a mixture), on the growth of strain Wilcox and its capacity to degrade BTEX compounds. The bacterium’s metal tolerance limit is defined as the highest concentration of a single metal or a mixture of metals at which complete degradation of all four BTEX compounds occurs in MSM containing 2.5 M NaCl. Above this concentration, BTEX degradation either did not occur or was partially degraded even after prolonged incubation, and cell viability was lost as confirmed by plating (with agar plates containing acetate as the sole carbon source). Our results confirmed that strain Wilcox did not survive in bottles with metal concentrations exceeding its tolerance limit.

#### Single Metal Tolerance

The tolerance of strain Wilcox to each of the ten different heavy metals (As^5+^, Mn^2+^, Cd^2+^, Zn^2+^, Cr^3+^, Pb^2+^, Se^4+^, Co^2+^, Ni^2+^, or Cu^2+^) was evaluated by adding each metal individually at varying concentrations to separate bottles containing 2.5 M NaCl and 25 − 35 μmol of each BTEX compound. Bottles were inoculated with 1 mL of strain Wilcox from the mother culture. Inoculated bottles that contained BTEX, 2.5 M NaCl, but no added metal(s) served as positive controls. Negative controls consisted of uninoculated, autoclaved bottles containing BTEX, a metal, and 2.5 M NaCl. Degradation of BTEX was monitored weekly until no further degradation of BTEX occurred or the tolerance limit was reached.

#### Multiple Metal Tolerance

The tolerance limit of the Wilcox strain for a mixture of metals was determined in serum bottles as described above. In this study, we evaluated the tolerance limits of strain Wilcox to a series of metal mixtures. Four different set of bottles were prepared to represent four different metal combination, (i) As^5+^, Mn ^2+^, Cd^2+^; (ii) As^5+^, Mn^2+^, Cd^2+^, Zn^2+^, Se^4+^; (iii) As^5+^, Mn^2+^, Cd^2+^, Zn^2+^, Se^4+^, Pb^2+^, Cr^3+^; and (iv) As^5+^, Mn^2+^, Cd^2+^, Zn^2+^, Se^4+^, Pb^2+^, Cr^3+^, Co^2+^, Ni^2+^, Cu^2+^. These mixtures represent the top 3, 5, and 7 most tolerated metals, as well as a mixture of all 10 metals (see Table 1). Each mixture was prepared by combining equimolar concentrations of the respective metals, and the tolerance limit was determined by assessing BTEX degradation in the presence of increasing concentrations of these metal mixtures. Uninoculated and metal-free bottles containing BTEX were included as controls.

**Table 1:** Tolerance Limits of Strain Wilcox to Individual Heavy Metals and Time Required for Complete Degradation of BTEX

heavy metal^a^	tolerance limit (mM)	tolerance limit (mg/L)	BTEX degradation (weeks)
Arsenic (As)	100	31200	2
Manganese (Mn)	100	16900	2
Cadmium (Cd)	12.5	2290	2
Zinc (Zn)	7	954	4
Selenium (Se)	3	789	3
Lead (Pb)	3	994	4
Chromium (Cr)	2	533	3
Cobalt (Co)	0.5	119	2
Nickel (Ni)	0.5	119	4
Copper (Cu)	0.25	43	5

a Individual stock solutions were prepared by dissolving each metal salt in deionized water. Tolerance limit was determined by growing strain Wilcox in serum bottles containing 49 mL of MSM with 2.5 M NaCl (14.5% NaCl) and 25−35 μmol of each BTEX compound. Individual metals were added to separate bottles at varying concentrations. Bottles were inoculated with 1 mL of strain Wilcox (∼5 × 10^5^ CFU) from the mother culture.

#### Effect of Salt Concentration on Metal Tolerance

The effect of NaCl concentration on heavy metal tolerance by strain Wilcox was evaluated in serum bottles prepared as described above. Three sets of bottles were prepared, each with a different NaCl concentration: 1, 2.5, and 3.5 M. To each set, As^5+^, Zn^2+^, Cd^2+^, or Cr^3+^ was added individually to a separate bottle. Bottles were inoculated with strain Wilcox and incubated at 30 °C. Inoculated bottles with BTEX but no metals served as positive controls, while uninoculated bottles with metals and BTEX served as negative controls. Headspace gas was sampled weekly, and BTEX degradation was monitored as described before.^25^

#### Multimetal Tolerance of Strain Wilcox in Raw Produced Water

For this experiment, we used a raw PW with a salinity of ∼0.72 M NaCl (4.2% NaCl) from the First Wilcox Formation in Payne County, OK. The experiment was conducted in two sets. In the first set, the original salinity was maintained. In the second, the PW salinity was raised from 0.72 to 2.5 M NaCl (4.2% to 14.5% NaCl) by adding more NaCl to understand metal tolerance at elevated salinity. The experiments were conducted in 160 mL serum bottles, each containing 49 mL of raw PW. The bottles were amended with a mixture of 10 metals, each metal at equimolar concentrations. Tolerance of strain Wilcox to multimetals was evaluated in the presence of increasing concentrations of all 10 metals. The bottles were sealed with Teflon-coated septa and aluminum caps. The bottles were spiked with 25−35 μmol of each BTEX compound and inoculated with 1 mL (approximately 5 × 10^5^ CFU) of strain Wilcox from the mother bottle. The bottles were incubated upside-down in the dark at 30 °C, and the degradation of BTEX was monitored biweekly using GC. Control groups were also set up similarly. Inoculated bottles with BTEX but no metals served as positive controls, while uninoculated bottles with metals and BTEX served as negative controls.

### Partitioning and Cellular Distribution of Heavy Metals in Strain Wilcox

Serum bottles with MSM containing NaCl and BTEX were prepared as described above, and bottles were amended with a sublethal concentration (ranging from 50% to 80% of the tolerance limit based on results in Table 1) of 10 different metals, each added to separate bottles (in triplicate) from the individual stock. The final concentration of the metal was as follows: Cu^2+^ at 0.125 mM, Ni^2+^ at 0.25 mM, Co^2+^ at 0.25 mM, Cr^3+^ at 1 mM, Se^4+^ at 2 mM, Pb^2+^ at 2 mM, Zn^2+^ at 5 mM, Cd^2+^ at 10 mM, As^5+^ at 80 mM, or Mn^2+^ at 80 mM. Bottles were inoculated with 1 mL of strain Wilcox from the mother bottle. After complete BTEX degradation, the concentration of metals in the culture supernatant (S1), loosely attached to the cell wall (S2), tightly attached to the cell wall (S3), and intracellular fraction (S4) was estimated using the procedure described previously with some modifications.^26,27^ When BTEX was completely degraded, the culture was centrifuged (10,000 rpm for 10 min at 4 °C) and the supernatant was decanted into a sterile 50 mL tube and designated as the extracellular fraction (S1). To estimate the loosely attached metal, the cell pellet was gently washed twice with 5 mL of sterile saline solution, the suspension was centrifuged (10,000 rpm for 10 min at 4 °C) and the supernatant was decanted into a sterile 10 mL tube (S2). To remove tightly attached metals, the cell pellet was treated with 10 mM sterile EDTA at room temperature with gentle agitation for 5 min.^28^ After centrifugation (10,000 rpm for 10 min at 4 °C), the supernatant (S3) was decanted into a sterile 10 mL tube. The cell pellet was then resuspended in 5 mL of sterile saline and the metal in this fraction corresponded to the internalized (intracellular) fraction (S4).^29^

The first three fractions (S1, S2, and S3) were acidified with concentrated trace metal grade HNO_3_ to a final concentration of 2% to solubilize metals. The cell pellet fraction (S4) was digested with concentrated HNO_3_ and heated at 85−90 °C for 2 h. The digest was then diluted with deionized water to achieve a 2% acid concentration, matching the matrix of the ICP-MS (Inductively Coupled Plasma Mass Spectrometry; Agilent Technologies) calibration standards. All samples were filtered (0.45 μm pore size) and metal concentrations in all four fractions were analyzed using ICP-MS.^30^ The percentage of initially added metal partitioned in each of the four fractions was then estimated (Table S2).

### Genome Annotation and Functional Characterization

We had previously sequenced and assembled a high-quality draft genome for *Modicisalibacter* sp. strain Wilcox, resulting in a 3.6 Mb genome comprising 3283 predicted protein-coding genes.^23,31^ Genome annotation was performed using both the Integrated Microbial Genomes (IMG) Annotation Pipeline v.5.0.19 ^32^ and the NCBI Prokaryotic Genome Annotation Pipeline v6.7.^33^ Predicted proteins were functionally characterized by mapping them to the Kyoto Encyclopedia of Genes and Genomes (KEGG) and Clusters of Orthologous Groups (COG) databases. KEGG orthology (KO) assignments were conducted using GhostKOALA v2.0,^34^ while COG assignments were performed with eggNOG mapper v2.1.12.^35^

#### Salt Tolerance and Heavy Metal Resistance Genes

To elucidate the genetic determinants of salt tolerance, the annotated genome was screened for homologues of known osmoregulatory and ion transport systems. These included genes encoding Na^+^/H^+^ antiporters, compatible solute synthesis enzymes, and transporters associated with osmotic stress adaptation.^36,37^ Genes associated with heavy metal resistance were identified by reviewing NCBI and IMG annotations for candidate resistance genes and gene clusters. These included genes encoding metal efflux pumps, metal transporters, metal reductases, P-type ATPases, and metallothioneins, which have been identified for many organisms in the literature. To confirm these findings, BLASTp searches were conducted against the UniProtKB reference proteomes and Swiss-Prot databases using default parameters.^38^

#### General Stress and Cross-Protection Mechanisms

To identify genetic determinants that could confer cross-protection to high salt and heavy metals, the annotated genome was systematically reviewed for hallmark general stress response genes. These included oxidative stress regulons and enzymes that help mitigate metal/salt-induced ROS, such as superoxide dismutases, catalases, and peroxidases; DNA-repair and protein quality-control modules, such as recABCD that mitigate collateral damage from salt/metal stress; biofilm formation, and EPS biosynthesis genes; and broad-specificity efflux systems such as resistance-nodulation-division (RND), Major facilitator superfamily (MFS), and ATP-Binding cassette (ABC) transporters implicated in tolerance to heavy metals and xenobiotics. The NCBI/IMG annotations were validated by BLASTp search against the UniProtKB reference proteomes and Swiss-Prot databases using default parameters.^38^

## Results and Discussion

Strain Wilcox was isolated from PW from the First Wilcox formation, Payne County, OK. We have previously reported the bacterium’s ability to degrade a variety of aliphatic and aromatic hydrocarbons under high salinity ranging from 3 to 24% NaCl.^23^ To obtain insights into its genetic potential, the genome was sequenced and assembled.^31^ This study reports on the bacterium’s capacity to survive and degrade BTEX under highly saline conditions and in the presence of heavy metals (individually or as a mixture) in laboratory microcosms. Most PW contains toxic hydrocarbons, heavy metals, and high salt, which makes it a hostile environment for microbial growth and function. We further analyzed the bacterium’s genome to understand the molecular basis of tolerance to heavy metals under high salinity conditions.

### Single Metal Tolerance under High Salinity Grown in MSM

We tested the tolerance limit of strain Wilcox to 10 different heavy metals individually at increasing concentrations in MSM containing 2.5 M NaCl. The strain exhibited a high tolerance to several metals, most at millimolar concentrations (Table 1). The strain’s tolerance to the metals in the following order of increasing concentration is Cu^2+^ < Ni^2+^ < Co^2+^ < Cr^3+^ < Pb^2+^ < Se^4+^ < Zn^2+^ < Cd^2+^ < Mn^2+^ < As^5+^. This shows that Cu^2+^, with a 0.25 mM tolerance limit, was the most toxic, while arsenic (As^5+^), with a tolerance limit of 100 mM, was the least toxic to the growth and survival of the bacterium. Linear regression analysis of the log-transformed BTEX degradation rate on metal concentration revealed a significant negative association for all the metals tested except Mn (*p*-value <0.05) (Table S1), consistent with an exponential, concentration-dependent inhibition over the ranges tested. However, within this overall trend, we also observed some nonlinear patterns. For Pb (0.5−2 mM), As (10−40 mM), and Mn (30−80 mM), BTEX degradation rate remained relatively constant within these subtolerance ranges.

Previous studies have shown that heavy metals inhibit microbial degradation of organic pollutants including hydrocarbons.^39^ While an increase in inhibition with increasing metal concentration is generally observed, other response patterns have also been reported including constant mild inhibition at subtolerance range.^40^ The stable degradation rate across a metal concentration interval likely reflects the microbial resistance mechanisms, such as biosorption, efflux, and intracellular sequestration, that reduce effective metal toxicity.^39,40^

It is also important to note that for certain metals, the tolerance limit for complete degradation varied among individual hydrocarbons. In the case of Pb^2+^, degradation of benzene, toluene, and ethylbenzene was inhibited at concentrations >3 mM, whereas xylene was fully degraded even at 5 mM (Figure S1). Similarly, in the case of Se^4+^, the tolerance limit was 3 mM for benzene and xylene, 5 mM for toluene, and 7 mM for ethylbenzene (Figure S2). The variation in the observed metal tolerance for different hydrocarbons by the same bacterium suggests that the enzymes involved have different sensitivities to heavy metals, with some being more susceptible to inhibition or functional disruption. In addition, in several experiments, BTEX degradation proceeded rapidly during the early incubation period but slowed or stopped before complete substrate removal. The observed loss of degradation over time indicates reduced metabolic activity at elevated metal concentrations, likely resulting from the saturation of metal tolerance mechanisms rather than substrate limitation.

Microbial tolerance to metals varies widely across taxa, including halophilic bacteria and archaea,^41–43^ and even a single strain can exhibit different tolerance levels to the same metal under varying growth conditions such as pH, substrate type, nutrient availability, temperature, redox state, or biofilm formation.^39,44^ Given the observed tolerance of strain Wilcox to elevated concentrations of many heavy metals that are typically found in PW, these results highlight the strain’s potential for PW treatment and support its robustness under polyextreme conditions.

### Effect of Salt Concentration on Metal Tolerance

The tolerance of strain Wilcox to As^5+^, Cd^2+^, Zn^2+^, or Cr^3+^ (Table 2) was evaluated in serum bottles as described above with MSM containing 1, 2.5, or 3.5 M NaCl and BTEX as the carbon source. Results showed that the strain needed more time to degrade BTEX with increasing concentration of NaCl and that incomplete degradation of BTEX was observed with Zn^2+^ or Cr^3+^ at 3.5 M NaCl (20.3% NaCl). Interestingly, in the case of Cd^2+^, incomplete degradation of BTEX occurred at a lower salinity of 1 M NaCl, whereas complete degradation was observed at higher concentrations of 2.5 and 3.5 M NaCl, suggesting that Cd^2+^ is more toxic to the cells at lower salinity.

**Table 2:** Effect of Salinity on Metal Tolerance by Strain Wilcox

^a^metal	metal concentration (mM)	NaCl (M)	^b^BTEX degradation
As^5+^	100	1	1 week
		2.5	2 weeks
		3.5	3 weeks
Cd^2+^	12.5	1	NCD
		2.5	2 weeks
		3.5	5 weeks
Zn^2+^	7	1	3 weeks
		2.5	5 weeks
		3.5	NCD
Cr^3+^	2	1	2 weeks
		2.5	3 weeks
		3.5	NCD

a Individual stock solutions were prepared by dissolving each metal salt in deionized water. The tolerance of strain Wilcox to As^5+^, Cd^2+^, Zn^2+^, or Cr^3+^ was evaluated by adding each metal individually to separate bottles containing varying NaCl concentrations.

b Time taken for complete degradation of BTEX at different salinity. NCD = No complete degradation of BTEX even after 6 weeks of incubation.

At 1 M NaCl, higher tolerance was observed for As^5+^, Zn^2+^, and Cr^3+^ compared to 2.5−3.5 M NaCl, possibly due to reduced energetic burdens for osmoregulation. When bacteria are faced with high salinity, they must actively pump ions or synthesize compatible solutes to maintain osmotic balance, and these processes demand energy in the form of ATP, ion potential, and the utilization of cellular resources.^36,37^ Similarly, dealing with heavy metal stress requires energy expenditure and the use of cellular resources for the active transport of these toxic ions out of the cell or for other detoxification mechanisms.^18^ When bacteria experience both high salinity and heavy metal stress simultaneously, the cells face a dual burden as they need to allocate energy and resources to counteract both stress components. Hence, our data support the idea that the presence of high salinity, requiring substantial energy for osmoregulation, may limit the cell’s ability to invest adequately in heavy metal tolerance mechanisms, and as a result, the bacteria may exhibit reduced tolerance to heavy metals under conditions of concurrent metals and osmotic stress. In contrast, tolerance to Cd^2+^ increased at higher salinity. An increase in salinity can lead to the formation of Cd−Cl complexes, which reduces the concentration of free Cd^2+^ and hence its toxicity.^45–47^ A study conducted using a halophilic *Pseudomonas* sp. reported decreased Cd^2+^ toxicity with increasing NaCl concentration and the authors surmise that this could be due to the increased efflux of Cd^2+^ at higher NaCl concentrations.^48^ Overall, the effect of NaCl on Cd^2+^ toxicity in microorganisms is complex and can vary depending on the specific bacterial species, NaCl concentration, and other environmental factors.

### Multimetal Tolerance of Strain Wilcox

The effect of multiple metals on the growth of strain Wilcox was evaluated in the presence of 3, 5, or 7 metals, as well as a mixture containing all 10 metals. The metals are combined based on increasing toxicity from As^5+^ to Cu^2+^ (see Table 1). Equimolar concentrations were used in each experiment. For example, in the mixture of As^5+^, Mn^2+^, and Cd^2+^, 7.5 mM means that the serum bottles contain 7.5 mM each of the three metals rather than the sum of the concentration of all three metals. In addition, for each metal combination, the concentration range tested was determined by the tolerance limit of the most toxic metal in that series. For instance, in the mixture of As^5+^, Mn^2+^, and Cd^2+^, Cadmium was the most toxic with a tolerance limit of 12.5 mM. Therefore, a concentration range that spans the lower and higher limits of Cd tolerance (7.5−20 mM) was tested. Degradation of BTEX was monitored across all metal combinations and concentrations (Figure 1a−d).

**Figure 1:**
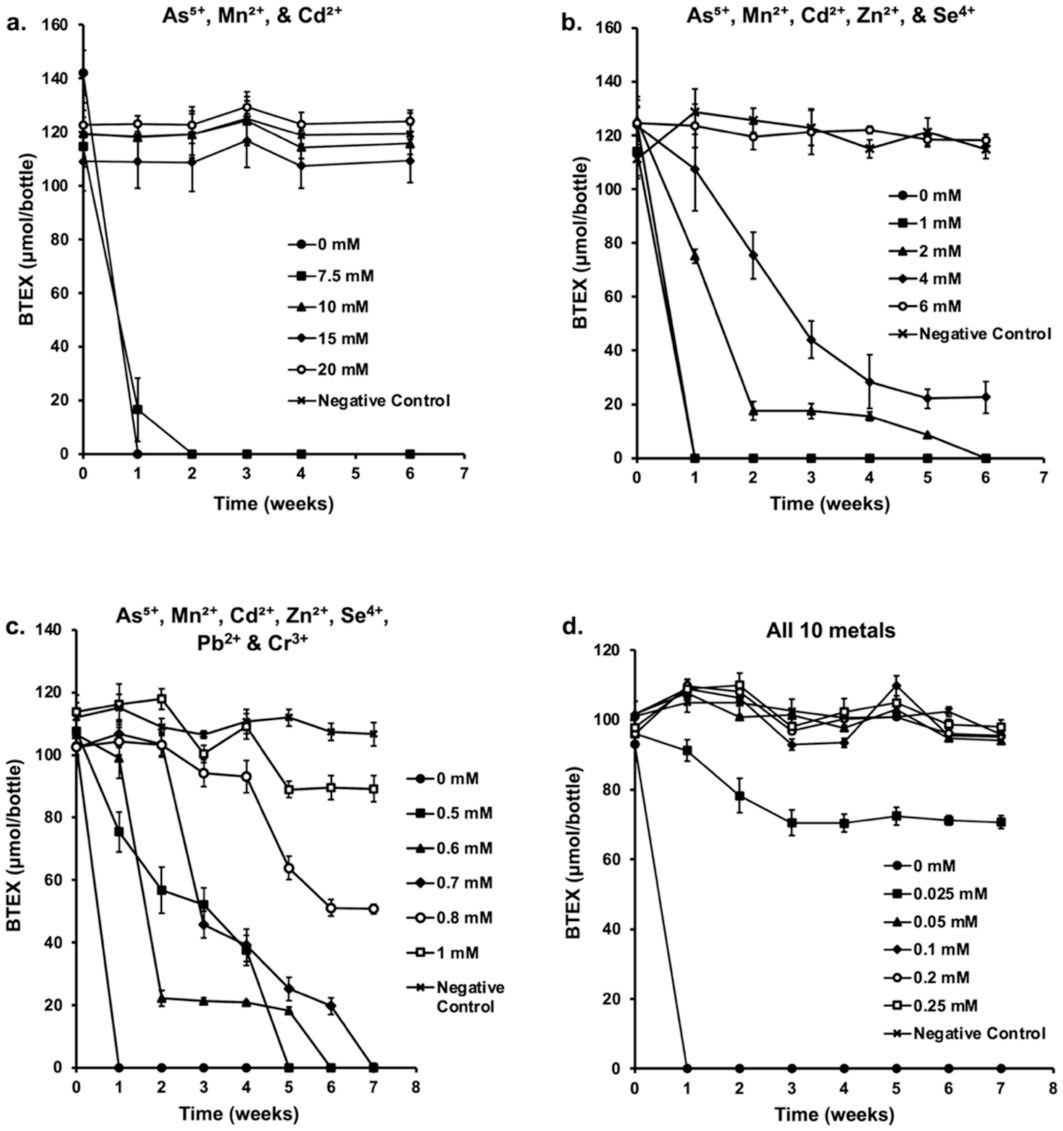
(a–d). The tolerance of strain Wilcox to multimetals. The Wilcox strain was cultivated in serum bottles containing MSM and 2.5 M NaCl. The bottles were amended with 25−35 μmol of each BTEX compound as the sole carbon source. To assess the impact of multimetals on BTEX degradation, separate experiments with four different metal combinations were carried out, (a) 3 metals: As^5+^, Mn^2+^, Cd ^2+^; (b) 5 metals: As^5+^, Mn^2+^, Cd^2+^, Zn^2+^, and Se^4+^; (c) 7 metals: As^5+^, Mn^2+^, Cd^2+^, Zn^2+^, Se^4+^, Pb,^2+^ Cr^3+^, and; (d) All 10 metals: As^5+^, Mn^2+^, Cd^2+^, Zn^2+^, Se^4+^, Pb^2+^, Cr^3+^, Co^2+^, Ni^2+^, and Cu^2+^. Equimolar concentration of each metal in four combinations was added to serum bottles. The tolerance was tested by adding an increasing concentration of the metal mixtures. Error bars indicate ± 1 standard deviations (*n* = 3).

Complete degradation of BTEX was achieved within 2 weeks in bottles containing a mixture of As^5+^, Mn^2+^, and Cd^2+^ at 7.5 mM, and no degradation was observed at higher concentrations (Figure 1a). Figure 1b shows the complete degradation of BTEX in bottles containing up to 2 mM As^5+^, Mn^2+^, Cd^2+^, Zn^2+^, or Se^4+^. At higher concentrations (3−4 mM), BTEX degradation remained incomplete even after 6 weeks of incubation and a similar trend was observed for mixtures of 7 and 10 metals, suggesting that metals exert higher toxicity when combined, compared to individual metal and that toxicity increases with the number of metal species present in a mixture. However, the absence of complete degradation does not imply that none of the compounds (benzene, toluene, ethylbenzene, or xylene) were degraded. In some cases, particularly combinations with Pb^2+^ or Se^4+^, toluene or ethylbenzene were degraded, whereas benzene or xylenes were partially degraded or not degraded at all, mirroring what was observed when the bacterium was exposed to Pb^2+^ or Se^4+^ alone (Figures S1 and S2).

Most PW contains a mixture of heavy metals dissolved from formation rocks. The geological composition of the subsurface formation varies by location, which influences both the type and concentration of heavy metals present in PW.^9,11^ These results therefore highlight the importance of evaluating multimetal tolerance for PW bioremediation as demonstrated in this study. They also caution against extrapolating single-metal tolerance data to complex field matrices, where such assumptions often lead to substantial discrepancies between laboratory and field outcomes.

Consistent with prior observations,^39,42^ multimetals can interact in complex ways to shape toxicity, leading to synergistic, additive, or antagonistic effects on microbial growth. The mixtures of 3, 7, and 10 metals (Figure 1a–d) show strong synergistic toxicity effects on the bacterium’s growth and BTEX degradation potential, with 40%, 65%, and 90% reduction in metal tolerance, respectively. However, in Figure 1b, the maximum toxicity by the 5 metals (As^5+^, Mn^2+^, Cd^2+^, Zn^2+^, and Se^4+^) is between 2 and 4 mM, which is closer to that of Se^4+^ toxicity when present singly, indicating no strong synergistic, additive, or antagonistic effects on the cells. Hence, the results imply that the toxicity of multimetal depends not only on the number of metals in the mixture, but also on what metals are present in the mixture. Exposure to a combination of metals may also induce additional molecular mechanisms not present when exposed to individual metals, which can enhance survival under multimetal stress conditions.^49^ Nonetheless, strain Wilcox growth and survival in the presence of high concentrations of different metal cocktails while simultaneously degrading hydrocarbons further confirm its potential for application in PW treatment.

### Multimetal Tolerance of Strain Wilcox Grown in Raw Produced Water

To validate the bacterium’s potential to tolerate heavy metals and degrade hydrocarbons, we replicated the multimetal tolerance experiment using raw PW from the Wilcox formation, Oklahoma. Because PW salinity varies widely by location, metal tolerance of strain Wilcox was assessed at two salinity levels (0.72 and 2.5 M). Figure 2a shows complete degradation of ∼107 μmol of BTEX at 0 and 0.025 mM metal concentrations within 2 and 4 weeks, respectively. However, at 0.05 mM, only partial degradation occurred (73%), and at higher concentrations, no degradation was observed in PW with ∼0.72 M NaCl (4.2% NaCl). However, when salinity was raised to 2.5 M NaCl (14.5% NaCl), complete inhibition of both growth and BTEX degradation occurred (Figure 2b), even in bottles without added metals. This inhibition is unexpected because the strain degrades BTEX at salinities up to 4 M NaCl (∼23% NaCl) in MSM.^23^ Therefore, we surmise the inhibition may have resulted from the combined effects of elevated salinity and other constituents (*e.g*., total dissolved solids, organics, surfactants, residual biocides, or inhibitors) in raw PW that may have exacerbated the toxicity.

**Figure 2:**
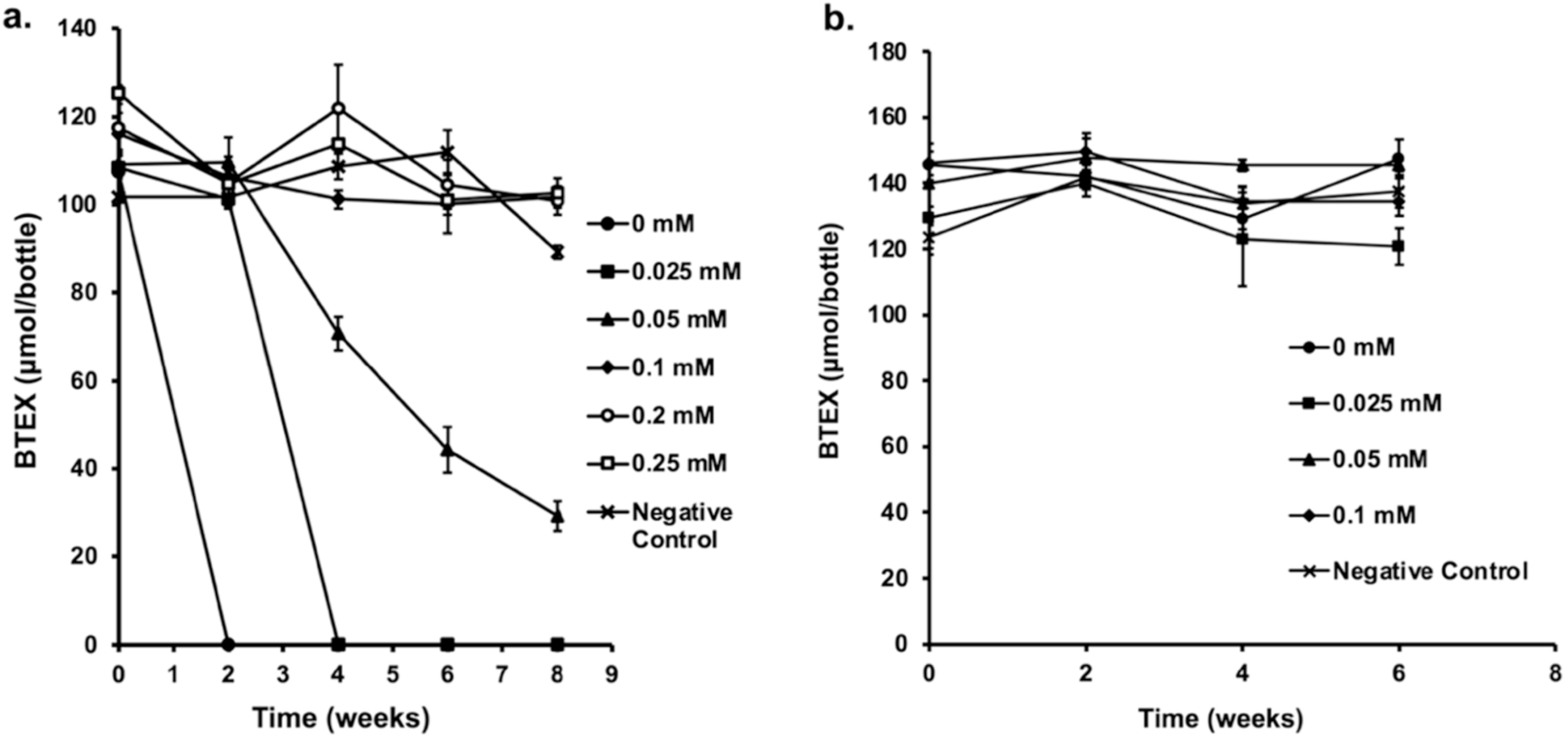
Tolerance of strain Wilcox to multimetals (As^5+^, Mn^2+^, Cd^2+^, Zn^2+^, Se^4+^, Pb^2+^, Cr^3+^, Co^2+^, Ni^2+^, Cu^2+^) in raw PW. The growth and BTEX degradation capacity of the strain were evaluated in raw PW at two salinity levels: (a) 0.72 M (4.2% w/v) and (b) 2.5 M NaCl (14.5% NaCl). Triplicate experimental bottles were prepared for each salinity condition (0.72 and 2.5 M NaCl). Each bottle was amended with a mixture of 10 metals each at equimolar concentrations. Tolerance of strain Wilcox to multimetals was evaluated in the presence of increasing concentrations of all 10 metals. The reported concentration values represent the concentration of each metal in the mix rather than the total combined metal concentration. Error bars indicate ± 1 standard deviation (*n* = 3).

### Partitioning and Cellular Distribution of Heavy Metals in Strain Wilcox

Microorganisms employ a diverse array of strategies for reducing the toxicity exerted by heavy metals. Some of the mechanisms include surface adsorption, intracellular accumulation, biotransformation, and active efflux.^18^ These mechanisms are crucial for microbial survival in metal-contaminated environments and play a significant role in the overall biogeochemical cycling of heavy metals. Understanding these processes will help develop bioremediation strategies and management of heavy metal pollution.

Results in Table 3 show that the cellular distribution of metals differs substantially depending on the type of metal. About 80 to 99% of added As^5+^, Cd^2+^, Se^4+^, Co^2+^, and Ni^2+^ were found in the supernatant, suggesting robust efflux and other transport mechanisms. Strain Wilcox’s genome contains *ars* operon with genes for arsenate/arsenite efflux and other P-type ATPase efflux genes, ABC transporters, and other transport mechanisms to expel As^5+^/As^3+^, Cd^2+^, Se^4+^, Co^2+^, and Ni^2+^ out of the cell. Extracellular binding (biosorption) of metals to cell walls and membranes is another effective strategy for metal sequestration outside the cell. Bacterial cell walls and extracellular polymeric substances possess negatively charged functional groups (*e.g*., carboxyl, phosphate, hydroxyl, and sulfhydryl) that act as binding sites for positively charged heavy metal ions. This biosorption can serve as a primary defense mechanism, preventing metals from entering the cell. Our results show that 44%, 19.7%, 81%, and 86% of the added Mn^2+^, Zn^2+^, Pb^2+^, and Cu^2+^, respectively, were firmly attached to the cell wall. In contrast, 29.7%, 59.3%, 17%, 18.7%, 96.4%, and 3.3% of the initially added Mn^2+^, Zn^2+^, Se^4+^, Pb^2+^, Cr^3+^, and Cu^2+^, respectively, were found intracellularly, suggesting a robust bioaccumulation strategy. Bacteria can accumulate metals within the cytoplasm in the form of granules or precipitates as a detoxification mechanism.^50^ Metal-binding proteins such as metallothioneins, glutathione (GSH), and metallochaperones can also help sequester heavy metal ions into nontoxic complexes, reducing their bioavailability and cellular toxicity.^50^

Overall, our study shows that strain was able to bioremediate (biosorption + bioaccumulation) 73.7% Mn^2+^, 79% Zn^2+^, 17.4% Se^4+^, 100% Pb^2+^, 98.6% Cr^3+^, or 90% Cu^2+^ of the initially added metal. The dual ability to degrade hydrocarbons and remove heavy metals under highly saline conditions therefore makes strain Wilcox a viable candidate for PW bioremediation of PW and other contaminated saline environments.

**Table 3:** Partitioning and Cellular Distribution of Heavy Metals in Strain Wilcox^a^

heavy metal	concentration of metal (mM)	extracellular (%)	loosely attached (%)	tightly attached (%)	intracellular (%)
Arsenic	80.0	99.6	0.2	0.1	0.1
Manganese	80.0	25.9	0.4	44.0	29.7
Cadmium	10.0	96.5	0.8	2.2	0.5
Zinc	5.0	20.6	0.3	19.7	59.3
Selenium	2.0	82.2	0.4	0.4	17.0
Lead	2.0	0.0	0.1	81.2	18.7
Chromium	1.0	1.1	0.3	2.2	96.4
Cobalt	0.25	96.9	1.4	0.0	1.7
Nickel	0.25	93.1	2.7	2.8	1.4
Copper	0.125	7.2	2.9	86.6	3.3

a Experiment was carried out in serum bottles containing 49 mL of MSM, 2.5 M NaCl (14.5%), 25−35 μmol of each BTEX compound, and a known amount of metal. Bottles were inoculated with 1 mL of strain Wilcox. Degradation of BTEX was monitored on a weekly basis and the experiment was terminated when all the added BTEX was consumed. The entire content of the bottles was centrifuged at 10,000 rpm. Partitioning of metals into culture supernatant (S1), loosely attached to the cell wall (S2), tightly attached to the cell wall (S3), and internalized fraction (S4) was determined using the protocol described in the Method Section. Percentage recovery of the initially added metal ranged from 95.1 − 104.3% (Table S2).

### Genes and Mechanisms for Heavy Metal Resistance

Genome mining shows that the bacterium encodes as many as 71 metal-relevant genes (Table 4) that contribute to its ability to withstand the toxicity of many heavy metals (Table 1). The bacterium exhibited high levels of tolerance to several metals when grown in MSM and salinity of 2.5 M NaCl, suggesting the robustness of the resistance mechanisms encoded by the bacterium’s genome. For example, the bacterium tolerated up to 100 mM As^5+^ while completely degrading the BTEX compounds in 2 weeks. Similarly, the strain withstood up to 100 mM Mn^2+^, 12.5 mM Cd^2+^, and 7 mM Zn^2+^ and degraded BTEX within 2−4 weeks. There are only a few examples of microorganisms that can tolerate such elevated levels of toxic arsenate. For example, Drewniak et al. have reported on the isolation of *Actinobacteria* and *Proteobacteria* exhibiting hypertolerance to about 500 mM arsenate.^51^ These microorganisms must possess robust detoxification mechanisms to survive such high levels of arsenate. Many microbes contain efflux systems that enable them to pump excess arsenate out of the cell.^52^

**Table 4:** Genes Involved in Heavy Metal Resistance by the *Modicisalibacter* sp. Strain Wilcox

NCBI gene ID	putative function	target metal (s)	mechanism
G7A98_RS00600^a^	Arsenate reductase ArsC_1	Arsenic	Reduction
G7A98_RS00610^a^	Arsenate reductase ArsC_2		
G7A98_RS13625^a^	Arsenate reductase ArsC_3		
G7A98_RS12425	Arsenate reductase ArsC_4		
G7A98_RS06245	Arsenate reductase ArsC_5		
G7A98_RS04095^a^	Arsenite efflux ATP-binding protein ArsA		Detoxification and efflux system
G7A98_RS13620^a^	Arsenical pump membrane protein ArsB		
G7A98_RS00590^a^	Arsenite efflux transporter Acr3		
G7A98_RS00595^a^	Arsenical resistance protein ArsH		
G7A98_RS02730	Arsenosugar biosynthesis glycosyltransferase_1		Sequestration and detoxification by biosynthesis of arsenosugars
G7A98_RS02725	Arsenosugar biosynthesis glycosyltransferase_2		
G7A98_RS13630	ArsR family transcriptional regulator_1		Transcriptional regulation
G7A98_RS06740	ArsR family transcriptional regulator_2		
G7A98_RS00605	ArsR family transcriptional regulator_3		
G7A98_RS00615	ArsJ-associated glyceraldehyde-3-phosphate dehydrogenase		Formation of organoarsenicals
G7A98_RS00620	Organoarsenical efflux MFS transporter ArsJ		Efflux of organoarsenicals
G7A98_RS01275	Cadmium/Lead-responsive transcriptional regulator CadR	Cadmium, Lead	Transcriptional regulation
G7A98_R813725	Cadmium/Zinc-transporting P-type ATPase	Cadmium, Zinc	Transmembrane transport
G7A98_RS11485^a^	Chromate transporter_1	Chromium	Efflux
G7A98_RS11490^a^	Chromate transporter_2		
G7A98_RS14415^a^	Cobalt/Magnesium efflux protein ApaG	Cobalt, Magnesium	Efflux
G7A98_RS01270^a^	Cobalt/Magnesium transporter CorA		Ion transport
G7A98_RS14890	Cobalt/Magnesium efflux protein CorC		Efflux
G7A98_RS02050^a^	Cobalt−zinc-cadmium efflux system protein czcD	Cobalt, Zinc, Cadmium	Efflux pump
G7A98_RS04750	Copper chaperone PCu(A)C	Copper	Metal trafficking/chaperone
G7A98_RS03570	Copper resistance protein NlpE		Sensing/Regulatory
G7A98_R502080^a^	Copper-exporting ATPase		Efflux
G7A98_R504620^a^	Copper-exporting P-type ATPase_1		
G7A98_RS04295^a^	Copper-exporting P-type ATPase_2		
G7A98_RS14505^a^	Multicopper oxidase		Metal Homeostasis
G7A98_RS02025	Copper/zing superoxide dismutase (SODC)	Copper, Zinc	Oxidative stress defense
G7A98_RS10815	Forric iron uptake transcriptional regulator	Iron	Transcriptional regulation
G7A98_RS02300	Iron ABC transporter permease_1		Uptake and transport system
G7A98_RS07305	Iron ABC transporter permease_2		
G7A98_RS02565	Iron ABC transporter permease_3		
G7A98_RS02565	Iron permease		
G7A98_RS02580	Iron uptake system protein EfeO		
G7A98_RS02575	Iron uptake transporter deferrochelatase/peroxidase subunit EfeB		Heme iron processing
G7A98_RS06445	Iron/manganese superoxide dismutase	Iron, Manganese	Oxidative stress defense
G7A98_RS04735	Manganese efflux pump MntP	Manganese	Efflux
G7A98_RS02775	Mercuric resistance operon regulatory protein MerR	Mercury	Transcriptional regulation
G7A98_RS02785^a^	Mercury(II) reductase MerA		Reduction
G7A98_RS02780	Mercury resistance system transport protein MerF		Efflux
G7A98_RS05315	Molybdate ABC transporter permease subunit	Molybdenum	Transport
G7A98_R805310	Molybdenum ABC transporter ATP-binding protein ModC		ATP-driven transport
G7A98_RS07240	Cation diffusion facilitator family transporter	Multiple	Efflux
G7A98_RS02075	Cation transporter		Ion transport
G7A98_RS02800	Cation-translocating P-type ATPase_1		Efflux
G7A98_RS12405	Cation-translocating P-type ATPase_2		Efflux
G7A98_RS04230	Divalent metal cation transporter MntH		Ion binding and transport
G7A98_RS16575	Periplasmic heavy metal sensor		Sensing/Signal transduction
G7A98_RS04820	Metallothionein		Metal sequestration
G7A98_RS02585	TetR/AcrR family transcriptional regulator_1		Transcriptional regulation
G7A98_RS09205	TetR/AcrR family transcriptional regulator_2		
G7A98_RS07345	TetR/AcrR family transcriptional regulator_3		
G7A98_RS06855	Hydrogenase maturation nickel metallochaperone HypA	Nickel	Uptake and transport system
G7A98_RS06395	Peptide/nickel transport system ATP-binding protein		
G7A98_RS06385	Peptide/nickel transport system permease protein_1		
G7A98_RS06390	Peptide/nickel transport system permease protein_2		
G7A98_RS06380	Peptide/nickel transport system substrate-binding protein		
G7A98_RS08255^a^	Lead, cadmium, and mercury-transporting ATPase	Lead, Cadmium, Zinc, Mercury	Efflux
G7A98_RS04770	Putative selenate ABC transporter substrate-binding protein	Selenium	Transport
G7A98_RS09010	Tellurite resistance protein B, TerB	Tellurium	Detoxification and efflux system
G7A98_RS10380^a^	Tellurite resistance protein TehA		
G7A98_RS07700	Tellurite resistance TerB family protein		
G7A98_RS11000	Uncharacterized conserved protein YaaN involved in tellurite resistance		
G7A98_RS02285^a^	Zinc ABC transporter substrate-binding protein	Zinc	Uptaka and transport
G7A98_RS08095^a^	Zinc transporter, ZntB		Efflux
G7A98_RS02270^a^	Zinc/manganese transport system ATP-binding protein	Zinc, Manganese	ATP-driven transport
G7A98_RS02275^a^	Zinc/manganese transport system permease protein		Membrane transport

a Genes reported in the previous study [Marsh et al.] from an initial screen.

Strain Wilcox’s high tolerance to arsenate is consistent with the presence of multiple resistance genes dedicated to maintaining a safe level of these metals in the cytoplasm. The genome analysis revealed that the bacterium encodes genes for well-known mechanisms of arsenic resistance, including 3 copies of transcriptional regulator (*ArsR*), 5 copies of arsenate reductase C (*ArsC*), 2 copies of arsenite membrane efflux pump (*ArsB*), and a copy of arsenate efflux ATP-binding protein (*ArsA*) genes. Some of these genes exist in operons (*ars* operon) and the core components include *arsR*, which regulates the operon, *arsC*, which allows the reduction of arsenate to arsenite, and *arsB*, which encodes a protein that forms a pump to expel arsenite.^53^ In addition, the genome contains 2 copies of genes for arsenosugar biosynthesis, glycosyltransferase, and one copy of organoarsenical efflux transporter (*ArsJ*), highlighting the bacterium’s potential to use multiple resistance mechanisms for maintaining safe levels of arsenate and arsenite.

Strain Wilcox also tolerated high levels of Mn (up to 100 mM). Manganese plays an important role as a cofactor for diverse enzymes involved in various metabolic processes, including oxidative stress resistance, primarily by breaking down reactive oxygen species.^54^ However, excessive Mn^2+^ can disrupt various physiological processes such as inhibiting enzyme activity and causing deficiencies in other metals. To avoid Mn^2+^ toxicity, bacteria maintain an optimal internal Mn^2+^ concentration with Mn importers and exporters. The strain Wilcox genome contains the *MntP* gene that codes for a manganese efflux pump that plays a critical role in removing excess manganese from the cell to maintain iron/manganese homeostasis and this in turn contributes to the bacterium’s ability to withstand oxidative stress.^55^

Strain Wilcox demonstrated high tolerance to both Cd^2+^ (up to 12 mM) and Zn^2+^ (up to 7 mM) when exposed to each metal individually, suggesting the strain’s ability to tolerate high levels of Zn^2+^ and Cd^2+^. Genome analysis shows that strain Wilcox contains a gene that codes for a cadmium transcriptional regulator (CadR) that regulates the transcription of a cadmium efflux pump, P-type ATPase (CadA). CadA is the most specific cadmium-binding protein, as the cadR promoter is strongly induced by Cd^2+^ and also weakly induced by Pb^2+^ and Zn^2+^.^56^ The genome also contains one gene for cobalt−zinc-cadmium efflux system protein (czcD), one copy of lead−cadmium-zinc-mercuric transporting ATPase, and one copy of copper/zinc superoxide dismutase (SODC). SODCs are responsible for limiting damage from reactive oxygen species from high levels of zinc.^57^ The presence of lead−cadmium-zinc-mercuric genes provides excellent protection from toxic metals like cadmium, zinc, and lead. They are crucial for maintaining cellular homeostasis by exporting heavy metals like zinc, cadmium, and lead from the intracellular environment. These ATPases utilize energy from ATP hydrolysis to move metal ions against their concentration gradients.^18^

In addition, the strain also grows and degrades BTEX in the presence of fairly high concentrations of some of the most toxic heavy metals such as Se^4+^ (up to 3 mM), Pb^2+^ (up to 3 mM), or Cr^3+^ (up to 2 mM). Though these metals can be harmful even at low levels, microbes have developed various mechanisms to tolerate and survive in the presence of these metals.^18,58^ Some anaerobes can use selenate (Se^6+^) and selenite (Se^4+^) as an electron acceptor for growth.^59^ Also, aerobic microorganisms can reduce Se^4+^ to elemental selenium (Se^0^) through aerobic reduction pathways using the glutathione reductase system and thioredoxin reductase system when exposed to higher concentrations of Se^4+^.^60^ The genome analysis also showed the presence of a putative selenate ABC transporter substrate-binding protein, indicating strain Wilcox might use the ABC transport system to maintain Se^4+^ homeostasis by transporting selenite out of the cell. The strain tolerates up to 2 mM chromite (Cr^3+^). Chromium exists in one of two stable oxidation states in the environment, chromate (Cr^6+^) and chromite (Cr^3+^); Cr^3+^ is less toxic compared to chromate. Microorganisms can enzymatically reduce Cr^6+^ to Cr^3+^ for tolerance.^61^ Our genome analysis predicted the presence of two chromate transporters. Chromate transporters are membrane proteins that play a vital role in the resistance of bacteria to chromate. These proteins act as chemiosmotic pumps, actively extruding chromate ions (CrO_4_^2−^) from the cell cytoplasm using the proton motive force.^62^ However, we do not know whether these transporters transport Cr^3+^ out of the cell. Reports also show that Cr^3+^ is detoxified by biosorption and bioaccumulation in the cells, resulting in less toxicity, and this is consistent with the high intracellular chromium (>96%) observed for strain Wilcox (Table 3).^63^

Among the 10 heavy metals tested, strain Wilcox exhibits relatively low tolerance to Co^2+^ (up to 0.5 mM), Ni^2+^ (up to 0.5 mM), and Cu^2+^ (up to 0.25 mM). At low concentrations, cobalt, copper, and nickel are essential transition metals that play crucial roles in various cellular functions. To survive the toxicity exerted by these metals at high concentrations, microbes must lower the cytoplasmic concentrations of these metals using various mechanisms. Our analysis shows that the genome contains genes for the cobalt/magnesium efflux protein ApaG, the cobalt/magnesium transporter CorA, the cobalt−zinc-cadmium efflux system protein, and czcD. All of these proteins play a role in the efflux of cobalt from the cytoplasm. Bacterial nickel transport systems are crucial for maintaining nickel homeostasis since Ni^2+^ is toxic at elevated levels.^64^ The genome also contains peptide/nickel transporter ATP-binding protein, peptide/nickel transport system substrate-binding protein, and peptide/nickel transport system permease proteins. These proteins are part of the ATP-binding cassette and other transport mechanisms, all targeted to expelling Ni^2+^ to the extracellular environment.^65^ Copper is an important and essential micronutrient required as a cofactor in multiple enzymic reactions such as electron transport, oxidative respiration, denitrification, methane oxidation etc.^66^ In the present study, the bacterium tolerated up to 0.025 mM Cu^2+^ and needed a relatively long time of 4 weeks to completely degrade BTEX. Copper homeostasis is necessary as a high concentration of this metal exerts a number of deleterious effects due to its high chemical reactivity, including mismetalation of iron−sulfur clusters and generation of highly reactive radical oxygen species.^67^ To reduce the toxicity and maintain copper homeostasis, strain Wilcox possesses several genes that code for several protein including copper chaperone, copper-exporting ATPase, copper resistance protein, copper-exporting P-type ATPase, multicopper oxidase, and copper−zinc superoxide dismutase that is essential for protecting cells from damage caused by oxidative stress.^66^

While the bacterium possesses several Cu-resistance genes and little to no Pb-specific resistance genes, the bacterium has a higher tolerance to Pb^2+^ compared to Cu^2+^, which turns out to be the most toxic metal among the tested metals. This suggests that the number of metal resistance genes is not the sole determinant of the level of the bacterium’s tolerance to a specific metal. It is possible that all genes needed for a function may not be present or the predicted genes may code for defective protein or function in other different pathway(s). Likewise, this observation highlights the potential impact of nonspecific genes or mechanisms for metal detoxification, as over 18% of the added Pb^2+^ was bioaccumulated by the bacterial cells compared to 3% of the added Cu^2+^. Future work will validate these gene-level predictions and elucidate the regulatory responses to distinct metals using RNA-seq

### Genes and Mechanisms for Salt Tolerance

Genomic analysis showed that strain Wilcox can utilize both “salt-in” (accumulation of K^+^ and exclusion of Na^+^) and “salt-out” (biosynthesis or accumulation of organic solutes) strategies to maintain osmotic balance (Table S3). In the ‘salt-in’ strategy, microorganisms maintain osmotic balance by accumulating high concentrations of inorganic salts, mainly K^+^, by expelling Na ^+^ out of the cells. High cytosolic K^+^ ions are less harmful to the cellular enzymes than high intracellular Na^+^ ions.^34^ Strain Wilcox is equipped with an array of alkali metal-cation and alkali metal-solute transporters, including sodium/solute symporters, sodium/proton antiporters, neurotransmitter/sodium symporters, potassium/proton antiporters, and amino acid-cation symporters, facilitating the expulsion of Na^+^ and retention of K^+^.^36^ Furthermore, the osmotic potential is also maintained through the uptake or synthesis of the organic compatible solute and excretion of salt from the cytoplasm to maintain a low concentration of Na^+^ in the cytoplasm. The bacterium possesses genes for synthesizing and transporting compatible solutes, such as glycine betaine, ectoine, hydroxyectoine, and choline, which serve as osmoprotectants.^37^ This dual strategy, encompassing both “salt-in” and “salt-out” mechanisms, enhances Wilcox’s adaptability, rendering it more flexible in responding to abrupt changes in salinity or osmotic conditions compared to organisms reliant solely on either mechanism.^36^

### Genes for Cross-Protection and Cotolerance Mechanisms for Salt and Heavy Metal Stress

Halophiles and salt-tolerant microorganisms are exposed to multiple environmental stresses, such as salinity, heavy metals, pH, oxidative stress, and toxic hydrocarbons. To adapt to such polyextreme environments, these microorganisms have evolved mechanisms that enable them to cope and survive multiple stresses.^68^

Genome analysis revealed several categories of genes that could contribute to strain Wilcox’s ability to tolerate both salt and heavy metal stress (Table S4). Genes involved in ion homeostasis and osmotic regulation, typically associated with salt tolerance, may also contribute to heavy metal resistance. Multiple oxidative stress response genes, including SODCs, peroxidase, ferredoxin, glutaredoxin, and thioredoxin, were annotated in the genome, which are important in neutralizing reactive oxygen species generated during both salinity and heavy metal exposure. Similar observations have been reported in halophytic plants, where salinity adaptation is associated with metal tolerance via ion transport and ROS scavenging systems,^69^ and the potential cross-tolerance due to shared mechanisms between salinity and metal stress has been suggested.^70^ The genome also encodes several chaperone proteins that help fold and refold other proteins, which can protect against protein damage caused by both salt and heavy metal stress.

Additional genes associated with general stress responses, such as DNA repair and biofilm formation, were also identified, suggesting broader protective mechanisms against both high salinity and heavy metal stress. DNA repair mechanisms help to counter the genotoxic effects of metal and salt stress, which primarily cause DNA damage via oxidative stress.^71^ Biofilm-associated extracellular polymeric substances (EPS) may sequester metal ions and provide a passive barrier, resulting in increased metal resistance in biofilms compared to planktonic cells.^44^ Similarly, the formation of biofilm and accumulation of exopolysaccharides by microbes under salt stress conditions have been reported.^72^

Importantly, the genome also harbors a diverse set of efflux genes, distinct from previously described ones. These efflux pumps likely play a contributory role in reducing intracellular metal and ion concentration and thereby promoting growth and BTEX degradation under high salinity and heavy metal conditions.

### Limitation and Challenges

While this study demonstrates the strong potential of *Modicisalibacter* sp. strain Wilcox for the bioremediation of metal- and hydrocarbon-contaminated saline PW, the experiments were conducted under controlled laboratory conditions using a defined synthetic medium. Under defined conditions, key variables such as salinity, metal concentrations, hydrocarbon composition, nutrient availability, temperature, and oxygen levels can be independently manipulated. This enables reproducible assessment of microbial degradation kinetics, metal resistance thresholds, stress responses, and metabolic pathways involved. Genomic analyses further provided insights into the genetic potential associated with salt tolerance, heavy metal tolerance, and stress-related cross-protection mechanisms. However, functional validation of gene expression, regulatory pathways, and enzyme activity under specific and combined stressors was beyond the scope of this study. As a result, laboratory systems are particularly valuable for strain screening, pathway elucidation, toxicity assessments, and optimization of operational parameters.

In contrast, field bioremediation occurs in chemically complex, heterogeneous, and dynamically evolving environments. Produced water in the field exhibits substantial spatial and temporal variability in salinity, metal speciation, hydrocarbon mixtures, pH, redox conditions, and the presence of inhibitory compounds. These factors can strongly influence microbial activity, the bioavailability of contaminants, and overall treatment performance. Moreover, field systems host diverse microbial communities whose interactions (both synergistic and antagonistic) can enhance or suppress hydrocarbon degradation and metal transformation processes in ways that are difficult to predict from single-strain laboratory experiments.

Another key distinction lies in the contaminant bioavailability. In laboratory systems, hydrocarbons and metals are often more accessible to microorganisms, whereas in field settings, they may be sequestered by complexation, sorption to solids, emulsification, or phase separation. Operational constraints, such as hydraulic retention time, mixing efficiency, temperature fluctuations, and nutrient delivery, further limit the direct transferability of laboratory-derived rates and efficiencies to field applications.

Despite these limitations, laboratory studies are indispensable for derisking field implementation. They provide foundational knowledge that guides the design of pilot- and field-scale systems, informs microbial selection or consortium development, and helps identify environmental thresholds beyond which bioremediation performance declines. Field bioremediation, in turn, serves as a critical validation step, integrating biological processes with site-specific geochemical and operational constraints.

Overall, laboratory bioremediation studies should be viewed as predictive tools that identify potential mechanisms and performance trends, rather than as direct surrogates for field behavior. Effective remediation of hydrocarbon- and heavy-metal-contaminated saline PW requires a tiered approach that progresses from laboratory experiments to pilot-scale validation and ultimately field deployment under realistic, site-specific conditions.

### Comparison with Existing Produced Water Treatment Technologies

A direct quantitative comparison of PW treatment technologies is challenging due to the large variability in salinity, metal composition, organic content, and operating conditions across studies. Therefore, Table S5 provides a qualitative comparison highlighting the scope of application, advantages, and limitations of bioremediation by microbes such as *Modicisalibacter* sp. strain Wilcox under laboratory conditions compared to field-scale physical, chemical, and electrochemical technologies.^73–82^ Unlike many conventional treatment approaches, strain Wilcox shows high potential to bioremediate both hydrocarbons and metals under hypersaline conditions, with minimal energy input, which makes it a promising organism for PW matrices that are challenging for conventional technologies.

## Conclusions

This study investigates the halophilic bacterium *Modicisalibacter* sp. strain Wilcox, which is notable for its ability to degrade a broad range of aliphatic and aromatic hydrocarbons across salinities ranging from 3% to 26% NaCl. These characteristics make it an excellent model for studying microbial adaptation and survival in polyextreme environments. In this work, we systematically evaluated the strain’s capacity to degrade BTEX compounds as sole carbon sources under varying salinity and heavy metal concentrations.

This study demonstrates the exceptional ability of strain Wilcox to tolerate heavy metals, degrade BTEX, and remove metals (75−99%) through biosorption and bioaccumulation under high-salinity conditions. These results highlight the strain’s potential for treating large volumes of PW for beneficial reuse following a desalination process.

## Supplementary Material



## Data Availability

The genome assembly was submitted to the IMG database and is available under the genome ID 2844586914. The unannotated scaffolds and SRA reads were also deposited in the NCBI GenBank with accession numbers WNXF00000000.1 and SRR10525457, respectively. The NCBI ReqSeq annotation is available under the ID GCF_010993675.1.

## Notes

Log−linear association between metal concentration and BTEX degradation rate (Table S1); partitioning and cellular distribution of heavy metals in strain Wilcox (ICP-MS data) (Table S2); the genetic determinants of salt tolerance in strain Wilcox (Table S3); genes for cross-protection and cotolerance mechanisms for salinity and heavy metal stress (Table S4); qualitative comparison of produced water treatment technologies, including bioremediation by *Modicisalibacter* sp. strain Wilcox (Table S5); degradation of benzene, toluene, ethylbenzene and xylenes in the presence of Pb^2+^ (Figure S1­(a–d)); degradation of benzene, toluene, ethylbenzene and xylenes in the presence of Se^4+^ (Figure S2­(a–d)) (PDF)

## References

[1] Basu, B. ; Roy, C. ; Saha, S. ; Tirkey, A. S. ; Biswas, R. ; Dey, A. ; Dhara, B. ; Mitra, A. K. Extremophiles to polyextremophiles: survival. In Extremophiles: A Paradox of Nature with Biotechnological Implications; Walter de Gruyter GmbH & Co KG, 2022; Vol. 1, pp 221–244 10.1515/9783110788488-011.

[2] Rampelotto P. H. (2013). Extremophiles
and extreme environments. Life.

[3] Mainka T., Weirathmüller D., Herwig C., Pflügl S. (2021). Potential
applications of halophilic microorganisms for biological treatment
of industrial process brines contaminated with aromatics. J. Ind. Microbiol. Biotechnol..

[4] Zhuang X., Han Z., Bai Z., Zhuang G., Shim H. (2010). Progress in decontamination
by halophilic microorganisms in saline wastewater and soil. Environ. Pollut..

[5] Oren A., Gurevich P., Azachi M., Henis Y. (1992). Microbial degradation
of pollutants at high salt concentrations. Biodegradation.

[6] Martínez-Espinosa R. M. (2024). Halophilic
archaea as tools for bioremediation technologies. Appl. Microbiol. Biotechnol..

[7] Shahrim N. A., Abounahia N. M., El-Sayed A. M. A., Saleem H., Zaidi S. J. (2023). An overview
on the progress in produced water desalination by membrane-based technology. J. Water Process Eng..

[8] Liu Y., Lu H., Li Y., Xu H., Pan Z., Dai P., Wang H., Yang Q. (2021). A review of treatment technologies
for produced water in offshore oil and gas fields. Sci. Total Environ..

[9] Amakiri K. T., Canon A. R., Molinari M., Angelis-Dimakis A. (2022). Review of
oilfield produced water treatment technologies. Chemosphere.

[10] Fathepure B. Z. (2014). Recent
studies in microbial degradation of petroleum hydrocarbons in hypersaline
environments. Front. Microbiol..

[11] ALL Consulting U.S. Produced Water Volumes and Management Practices in 2021Ground Water Protection Council, 2022. www.gwpc.org.

[12] Ali H., Khan E. (2018). What are heavy metals?
Long-standing controversy over the scientific
use of the term “heavy metals”—proposal of a
comprehensive definition. Toxicol. Environ.
Chem..

[13] Gan Y., Wang L., Yang G., Dai J., Wang R., Wang W. (2017). Multiple factors
impact the contents of heavy metals in vegetables
in high natural background area of China. Chemosphere.

[14] Sonone S. S., Jadhav S., Sankhla M. S., Kumar R. (2020). Water contamination
by heavy metals and their toxic effect on aquaculture and human health
through food chain. Lett. Appl. NanoBioSci.

[15] Oves M., Khan M. S., Qari A. H., Felemban M. N., Almeelbi T. (2016). Heavy metals:
biological importance and detoxification strategies. J. Bioremediat. Biodegrad..

[16] Tchounwou, P. B. ; Yedjou, C. G. ; Patlolla, A. K. ; Sutton, D. J. Heavy metal toxicity and the environment. In Molecular, Clinical and Environmental Toxicology; Environmental Toxicology, 2012; Vol. 3, pp 133–164. 10.1007/978-3-7643-8340-4_6. PMC4144270 22945569

[17] Elnabi M. K. A., Elkaliny N. E., Elyazied M. M., Azab S. H., Elkhalifa S. A., Elmasry S., Mouhamed M. S., Shalamesh E. M., Alhorieny N. A., Elaty A. E. A., Elgendy I. M. (2023). Toxicity
of heavy metals and recent advances in their removal: a review. Toxics.

[18] Nnaji N. D., Anyanwu C. U., Miri T., Onyeaka H. (2024). Mechanisms of heavy
metal tolerance in bacteria: a review. Sustainability.

[19] Chandrangsu P., Rensing C., Helmann J. D. (2017). Metal homeostasis and resistance
in bacteria. Nat. Rev. Microbiol..

[20] Jamal M. T., Pugazhendi A. (2018). Degradation
of petroleum hydrocarbons and treatment
of refinery wastewater under saline condition by a halophilic bacterial
consortium enriched from marine environment (Red Sea), Jeddah, Saudi
Arabia. 3 Biotech.

[21] Duraisamy P., Sekar J., Arunkumar A. D., Ramalingam P. V. (2020). Kinetics
of phenol biodegradation by heavy metal tolerant rhizobacteria *Glutamicibacter nicotianae* MSSRFPD35 from distillery
effluent contaminated soils. Front. Microbiol..

[22] Khalil C. A., Prince V. L., Prince R. C., Greer C. W., Lee K., Zhang B., Boufadel M. C. (2021). Occurrence
and biodegradation of
hydrocarbons at high salinities. Sci. Total
Environ..

[23] Marsh W. S., Heise B. W., Krzmarzick M. J., Murdoch R. W., Fathepure B. Z. (2021). Isolation
and characterization of a halophilic *Modicisalibacter* sp. strain Wilcox from produced water. Sci.
Rep..

[24] Dalvi S., Youssef N. H., Fathepure B. Z. (2016). Microbial
community structure analysis
of a benzoate-degrading halophilic archaeal enrichment. Extremophiles.

[25] Nicholson C. A., Fathepure B. Z. (2004). Biodegradation of benzene by halophilic
and halotolerant
bacteria under aerobic conditions. Appl. Environ.
Microbiol..

[26] Bates S. S., Tessier A., Campbell P. G., Buffle J. (1982). Zinc adsorption and
transport by *Chlamydomonas varuiabilis* and *Scenedesmus subspicatus* (Chlorophyceae)
grown in semicontinuous culture 1. J. Phycol..

[27] Smiejan A., Wilkinson K. J., Rossier C. (2003). Cd bioaccumulation by a freshwater
bacterium, *Rhodospirillum rubrum*. Environ. Sci. Technol..

[28] Chandra P., Singh D. P. (2014). Removal of Cr­(VI)
by a halotolerant bacterium *Halomonas* sp. CSB 5 isolated
from Sambhar salt lake, Rajasthan
(India). Cell. Mol. Biol..

[29] Yuliani D., Morishita F., Imamura T., Ueki T. (2024). Vanadium accumulation
and reduction by vanadium-accumulating bacteria isolated from the
intestinal contents of *Ciona robusta*. Mar. Biotechnol..

[30] U.S. EPA. . Method 6020B (SW-846): Inductively Coupled Plasma−Mass Spectrometry U.S. Environmental Protection Agency: Washington, DC; 2014. www.epa.gov.

[31] Ajagbe D., Marsh W., Murdoch R., Fathepure B. (2025). Draft genome
sequence of *Modicisalibacter* sp. strain Wilcox isolated
from produced water. Microbiol. Resour. Announc..

[32] Chen I.-M., Chu K., Palaniappan K., Pillay M., Ratner A., Huang J., Huntemann M., Varghese N., White J. R., Seshadri R., Smirnova T., Kirton E., Jungbluth S. P., Woyke T., Eloe-Fadrosh E. A., Ivanova N. N., Kyrpides N. C. (2019). IMG/M v.
5.0: an integrated data management and comparative analysis system
for microbial genomes and microbiomes. Nucleic
Acids Res..

[33] Tatusova T., DiCuccio M., Badretdin A., Chetvernin V., Nawrocki E. P., Zaslavsky L., Lomsadze A., Pruitt K. D., Borodovsky M., Ostell J. (2016). NCBI prokaryotic genome annotation
pipeline. Nucleic Acids Res..

[34] Kanehisa M., Sato Y., Morishima K. (2016). BlastKOALA
and GhostKOALA: KEGG tools
for functional characterization of genome and metagenome sequences. J. Mol. Biol..

[35] Cantalapiedra C. P., Ana H. P., Ivica L., Peer B., Jaime H. C. (2021). eggNOG-mapper
v2: functional annotation, orthology assignments, and domain prediction
at the metagenomic scale. Mol. Biol. Evol..

[36] Csonka L. N. (1989). Physiological
and genetic responses of bacteria to osmotic stress. Microbiol. Rev..

[37] Gunde-Cimerman N., Plemenitaš A., Oren A. (2018). Strategies of adaptation of microorganisms
of the three domains of life to high salt concentrations. FEMS Microbiol. Rev..

[38] Martin M. J., Orchard S., UniProt Consortium (2023). UniProt: the universal
protein knowledgebase
in 2023. Nucleic Acids Res..

[39] Feng J. R., Ni H. G. (2024). Effects of heavy metals and metalloids on the biodegradation of organic
contaminants. Environ. Res..

[40] Gopinath K. P., Kathiravan M. N., Srinivasan R., Sankaranarayanan S. (2011). Evaluation
and elimination of inhibitory effects of salts and heavy metal ions
on biodegradation of Congo red by *Pseudomonas* sp.
mutant. Bioresour. Technol..

[41] Voica D. M., Bartha L., Banciu H. L., Oren A. (2016). Heavy metal resistance
in halophilic Bacteria and Archaea. FEMS Microbiol.
Lett..

[42] Matarredona L., Zafrilla B., Camacho M., Bonete M. J., Esclapez J. (2024). Understanding
the tolerance of halophilic archaea to stress landscapes. Environ. Microbiol. Rep..

[43] Rosas-Ramírez J. R., Isaac-Olivé K., Moreno-Pérez M.
P., Manzanares-Leal G. L., Serment-Guerrero J. H., Sandoval-Trujillo Á.
H., Ramírez-Durán N. (2023). Identification
of halophilic bacteria tolerant to heavy metals. Rev. Int. Contam. Ambient..

[44] Teitzel G. M., Parsek M. R. (2003). Heavy metal resistance of biofilm and planktonic *Pseudomonas aeruginosa*. Appl. Environ.
Microbiol..

[45] Engel D. W., Fowler B. A. (1979). Factors influencing
cadmium accumulation and its toxicity
to marine organisms. Environ. Health Perspect..

[46] Cheng M., Wang A., Liu Z., Gendall A. R., Rochfort S., Tang C. (2018). Sodium chloride decreases
cadmium accumulation and changes the response
of metabolites to cadmium stress in the halophyte *Carpobrotus
rossii*. Ann. Bot..

[47] Wildgust M.
A., Jones M. B. (1998). Salinity
change and the toxicity of the free cadmium
ion [Cd^2+^(aq)] to *Neomysis integer* (Crustacea: Mysidacea). Aquat. Toxicol..

[48] Kobayashi T., Tokunaga H., Seki K., Onishi H. (1993). Effects of salt concentration
on the intracellular cadmium content of and the cadmium uptake by
a highly cadmium-tolerant, moderately halophilic *Pseudomonas* sp. Biosci., Biotechnol., Biochem..

[49] Vandenbrouck T., Soetaert A., van der Ven K., Blust R., De Coen W. (2009). Nickel and
binary metal mixture responses in *Daphnia magna*: molecular fingerprints and (sub)­organismal effects. Aquat. Toxicol..

[50] Bazzi W., Fayad A. G. A., Nasser A., Haraoui L. P., Dewachi O., Abou-Sitta G., Nguyen V. K., Abara A., Karah N., Landecker H., Knapp C. (2020). Heavy metal toxicity
in armed conflicts potentiates AMR in *A. baumannii* by selecting for antibiotic and heavy metal co-resistance mechanisms. Front. Microbiol..

[51] Drewniak L., Styczek A., Majder-Lopatka M., Sklodowska A. (2008). Bacteria,
hypertolerant to arsenic in the rocks of an ancient gold mine, and
their potential role in dissemination of arsenic pollution. Environ. Pollut..

[52] Lin Y. F., Walmsley A. R., Rosen B. P. (2006). An arsenic
metallochaperone for an
arsenic detoxification pump. Proc. Natl. Acad.
Sci. U.S.A..

[53] Nanda M., Kumar V., Sharma D. K. (2019). Multimetal tolerance mechanisms in
bacteria: the resistance strategies acquired by bacteria that can
be exploited to clean up heavy metal contaminants from water. Aquat. Toxicol..

[54] Juttukonda L. J., Skaar E. P. (2015). Manganese homeostasis and utilization
in pathogenic
bacteria. Mol. Microbiol..

[55] Waters L. S. (2020). Bacterial
manganese sensing and homeostasis. Curr. Opin.
Chem. Biol..

[56] Lee S. W., Glickmann E., Cooksey D. A. (2001). Chromosomal locus for cadmium resistance
in *Pseudomonas putida* consisting of a cadmium-transporting
ATPase and a MerR family response regulator. Appl. Environ. Microbiol..

[57] Niu A., Bian W. P., Feng S. L., Pu S. Y., Wei X. Y., Yang Y. F., Song L. Y., Pei D. S. (2021). Role of manganese
superoxide dismutase (Mn-SOD) against Cr­(III)-induced toxicity in
bacteria. J. Hazard. Mater..

[58] Oleńska E., Małek W., Swiecicka I., Wójcik M., Thijs S., Vangronsveld J. (2025). Bacteria under
metal stress—molecular
mechanisms of metal tolerance. Int. J. Mol.
Sci..

[59] Gonzalez-Gil G., Lens P. N., Saikaly P. E. (2016). Selenite reduction by anaerobic microbial
aggregates: microbial community structure and proteins associated
with the produced selenium spheres. Front. Microbiol..

[60] Martínez F. G., Moreno-Martin G., Pescuma M., Madrid-Albarrán Y., Mozzi F. (2020). Biotransformation
of selenium by lactic acid bacteria: formation
of seleno-nanoparticles and seleno-amino acids. Front. Bioeng. Biotechnol..

[61] Alam M. Z., Malik A. (2008). Chromate resistance, transport and
bioreduction by *Exiguobacterium* sp. ZM-2 isolated
from agricultural soil irrigated with tannery
effluent. J. Basic Microbiol..

[62] Chromiková Z., Chovanová R. K., Tamindžija D., Bártová B., Radnović D., Bernier-Latmani R., Barák I. (2022). Implantation
of *Bacillus pseudomycoides* chromate
transporter increases chromate tolerance in *Bacillus
subtilis*. Front. Microbiol..

[63] Tang X., Huang Y., Li Y., Wang L., Pei X., Zhou D., He P., Hughes S. S. (2021). Study on detoxification
and removal mechanisms of hexavalent chromium by microorganisms. Ecotoxicol. Environ. Saf..

[64] Macomber L., Hausinger R. P. (2011). Mechanisms
of nickel toxicity in microorganisms. Metallomics.

[65] Eitinger T., Mandrand-Berthelot M.-A. (2000). Nickel
transport systems in microorganisms. Arch. Microbiol..

[66] Hyre A., Casanova-Hampton K., Subashchandrabose S. (2021). Copper homeostatic mechanisms and
their role in the virulence of *Escherichia coli* and *Salmonella enterica*. EcoSal Plus.

[67] Argüello J. M., Raimunda D., Padilla-Benavides T. (2013). Mechanisms
of copper homeostasis
in bacteria. Front. Cell. Infect. Microbiol..

[68] DasSarma S., DasSarma P., Laye V. J., Schwieterman E. W. (2020). Extremophilic
models for astrobiology: haloarchaeal survival strategies and pigments
for remote sensing. Extremophiles.

[69] Nikalje G. C., Suprasanna P. (2018). Coping with
metal toxicity—cues from halophytes. Front. Plant Sci..

[70] Hamed K. B., Ellouzi H., Talbi O. Z., Hessini K., Slama I., Ghnaya T., Bosch S. M., Savouré A., Abdelly C. (2013). Physiological response of halophytes to multiple stresses. Funct. Plant Biol..

[71] Rath S., Das S. (2023). Oxidative stress-induced
DNA damage and DNA repair mechanisms in
mangrove bacteria exposed to climatic and heavy metal stressors. Environ. Pollut..

[72] Qurashi A. W., Sabri A. N. (2012). Bacterial exopolysaccharide and biofilm
formation stimulate
chickpea growth and soil aggregation under salt stress. Braz. J. Microbiol..

[73] Priyadarshini M., Das I., Ghangrekar M. M., Blaney L. (2022). Advanced oxidation
processes: Performance, advantages, and scale-up of emerging technologies. J. Environ. Manage..

[74] Oturan M. A., Aaron J. J. (2014). Advanced oxidation processes in water/wastewater
treatment:
principles and applications. A review. Crit.
Rev. Environ. Sci. Technol..

[75] Fakhru’l-Razi A., Pendashteh A., Abdullah L. C., Biak D. R. A., Madaeni S. S., Abidin Z. Z. (2009). Review
of technologies for oil and gas produced water
treatment. J. Hazard. Mater..

[76] Igunnu E. T., Chen G. Z. (2014). Produced water treatment
technologies. Int. J. Low-Carbon Technol..

[77] Shahrim N. A. A., Abounahia N. M., El-Sayed A. M. A., Saleem H., Zaidi S. J. (2023). An overview
on the progress in produced water desalination by membrane-based technology. J. Water Process Eng..

[78] Pawar R., Zhang Z., Vidic R. D. (2022). Laboratory and pilot-scale
studies
of membrane distillation for desalination of produced water from Permian
Basin. Desalination.

[79] Li, F. ; Wu, X. ; Wu, J. ; Xu, X. ; Ma, S. In Kinetic Study of Adsorption of Oil from Oilfield Produced Water using Modified Porous Ceramics Filtration Media in Column Mode, 2nd International Conference on Bioinformatics and Biomedical Engineering; IEEE, 2008; pp 2745–2748.

[80] Doyle, D. H. ; Brown, A. B. In Field Test of Produced Water Treatment with Polymer Modified Bentonite, SPE Rocky Mountain Petroleum Technology Conference/Low Permeability Reservoirs Symposium; OnePetro, 1997.

[81] Li G., An T., Chen J., Sheng G., Fu J., Chen F., Zhang S., Zhao H. (2006). Photoelectrocatalytic decontamination
of oilfield produced wastewater containing refractory organic pollutants
in the presence of high concentration of chloride ions. J. Hazard. Mater..

[82] Ebadi S., Ghasemipanah K., Alaie E., Rashidi A., Khataee A. (2021). COD removal
from gasfield produced water using photoelectrocatalysis process on
coil type microreactor. J. Ind. Eng. Chem..

