# Supplementary material for: Bioremediation of Produced Water by a Polyextremophilic, Heavy-Metal-Resistant Modicisalibacter sp. Strain Wilcox
Source: ACS ES T Water. 2026 Mar 26;6(4):2364–77. doi: 10.1021/acsestwater.5c01373 (PMC13077682; doi:10.1021/acsestwater.5c01373)
Supplement: Supplementary file 1 [file ew5c01373_si_001.pdf]

Supplementary Information

Bioremediation of Produced Water by a Polyextremophilic, Heavy-Metal-Resistant *Modicisalibacter* sp. strain Wilcox

Damilare Ajagbe[1], Mark Krzmarzick[2], Babu Fathepure[1*]

[1]Department of Microbiology and Molecular Genetics, Oklahoma State University, Stillwater, OK, 74078
[2]Department of Civil and Environmental Engineering, Oklahoma State University, Stillwater, OK, 74078
*Corresponding author: babu.fathepure@okstate.edu

Table S1: Log-linear association between metal concentration and BTEX degradation rate.

Metal	Slope	P-value
Cu	-2.41	0.034
Co	-2.31	0.031
Ni	-2.07	0.033
Cr	-1.64	0.003
Pb	-0.53	0.033
Se	-0.28	0.001
Zn	-0.40	0.003
Cd	-0.13	0.001
Mn	-0.02	0.149
As	-0.02	0.046

For each metal, we fit ln(BTEX degradation rate) ~ concentration using ordinary least squares. Negative slopes indicate concentration-dependent inhibition. P-values < 0.05 denote a significant association across the tested concentration range. All metals showed negative slopes with $p < 0.05$ except Mn.

Degradation of Benzene, Toluene, Ethylbenzene and Xylenes in the presence of Pb^{2+}

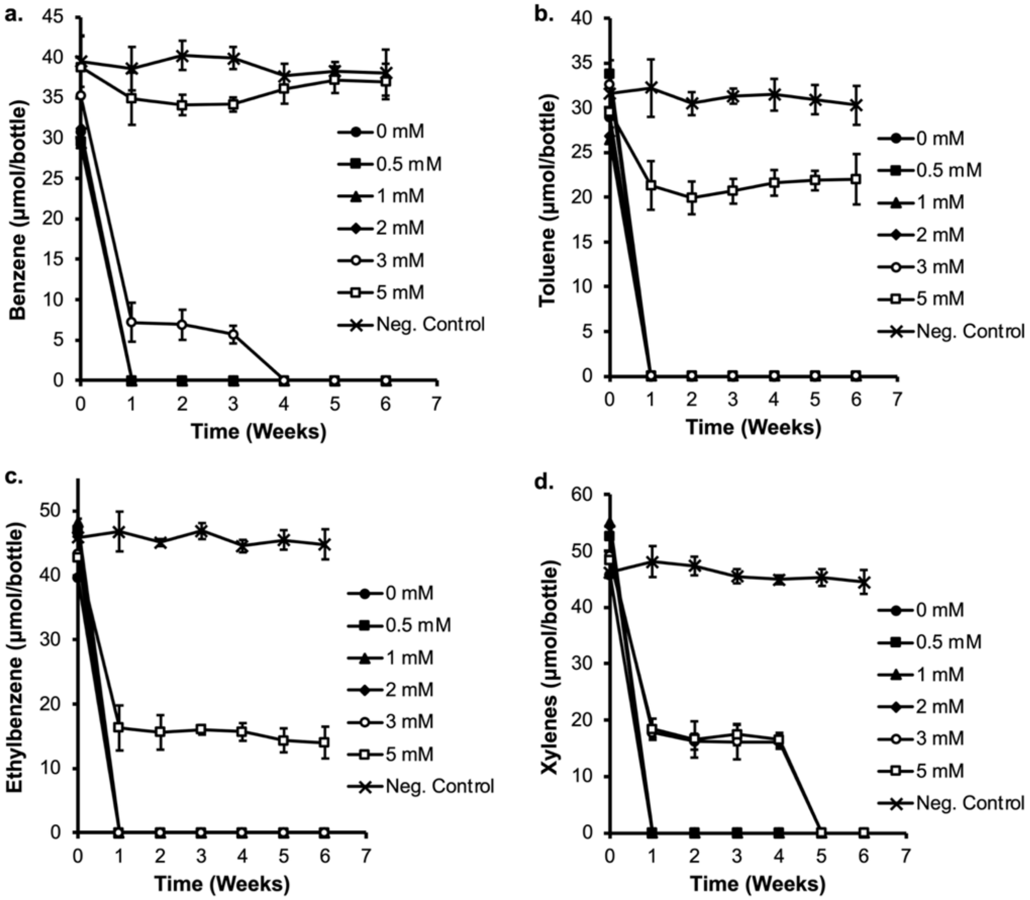

Fig. S1 (a–d). Degradation of (a) benzene, (b) toluene, (c) ethylbenzene, and (d) xylenes in the presence of increasing concentration of **Pb^{2+}**. "Neg. Control" denotes abiotic controls. Error bars indicate ±1 standard deviations (n = 3). Benzene degradation was completely inhibited at 5 mM Pb^{2+}; Toluene and Ethylbenzene showed only partial degradation at 5 mM; Xylenes were completely degraded at 5 mM.

Degradation of Benzene, Toluene, Ethylbenzene and Xylenes in the presence of Se^{4+}

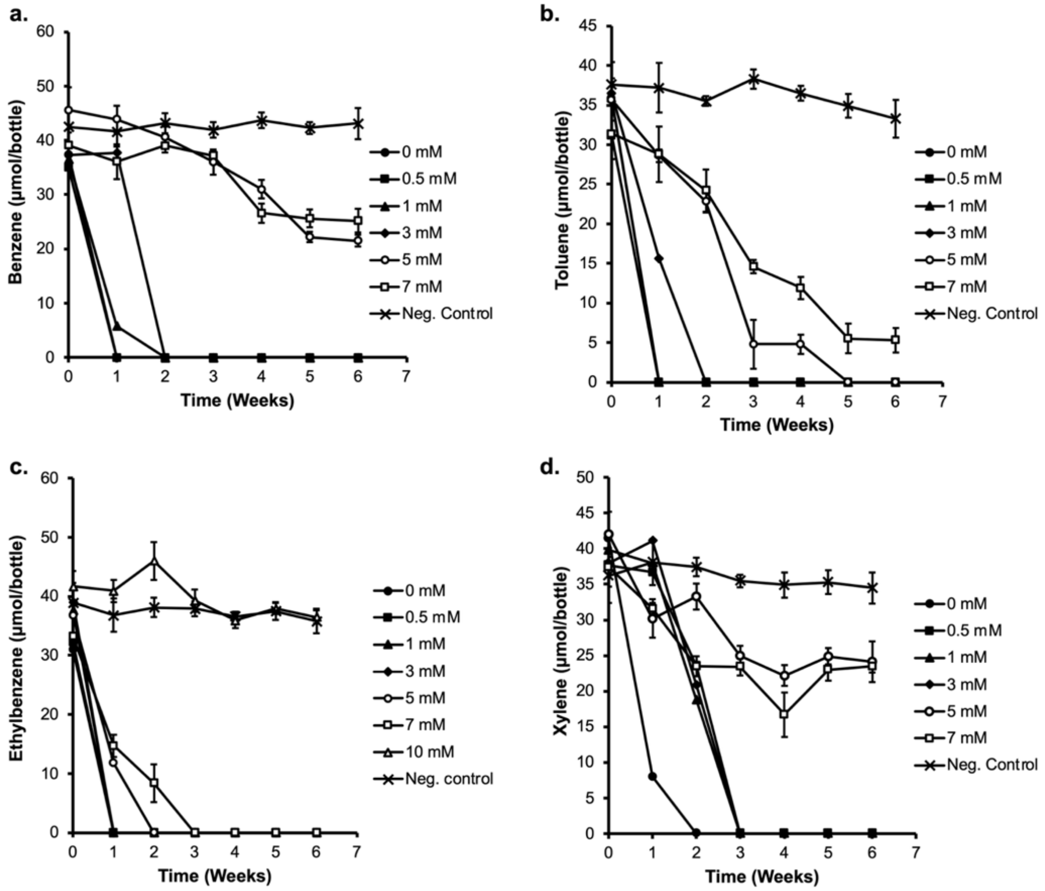

Fig. S2 (a–d). Degradation of (a) benzene, (b) toluene, (c) ethylbenzene, and (d) xylenes in the presence of increasing concentration of **Se^{4+}**.“Neg. Control” denotes abiotic controls. Error bars indicate ±1 standard deviations (n = 3). Benzene and Xylenes showed only partial degradation (≤ 50%) at ≥ 5 mM; Toluene degraded up to ~85% at 7 mM; Ethylbenzene was completely degraded at 7 mM and no degradation at 10 mM.

Table S2: Partitioning and cellular distribution of heavy metals in strain Wilcox

Heavy Metal	Metal Concentration (mM)	Estimated Metal Concentration (mg/L)	Extracellular (mg/L)	Loosely Attached (mg/L)	Tightly Attached (mg/L)	Intracellular (mg/L)	Total Metal Recovered (mg/L)	% Recovery
Arsenic	80	5990	5880	11.8	5.9	5.9	5900	98.5
Manganese	80	4390	1160	18.0	1980	1340	4490	102.4
Cadmium	10	1120	1050	8.8	24.2	5.5	1090	97.6
Zinc	5	327	64.2	0.9	61.4	185	310	95.2
Selenium	2	158	124	0.6	0.6	25.6	150	95.1
Lead	2	414	0.02	0.4	351	80.5	432	104.3
Chromium	1	52	0.6	0.2	1.2	51.5	53.4	102.8
Cobalt	0.25	14.7	14.0	0.2	0.0	0.2	14.4	98.0
Nickel	0.25	14.7	13.2	0.4	0.4	0.2	14.2	96.3
Copper	0.125	7.94	0.6	0.2	7.0	0.3	8.1	102.1

% Recovery = (Sum of the four extracted fractions / Initial metal concentration) × 100.

Table S3. The genetic determinants of salt tolerance in strain Wilcox

NCBI Gene ID	Putative Function	Mechanism	Functional Category
G7A98_RS11000	Na+/H+ antiporter	Na+/H+ antiporters	Salt-in strategy
G7A98_RS04485	Na+/H+ antiporter subunit A		
G7A98_RS14800	Na+/H+ antiporter		
G7A98_RS02100	Na+/H+ antiporter		
G7A98_RS14920	Na+/H+ antiporter NhaC		
G7A98_RS04460	Na+/H+ antiporter subunit G		
G7A98_RS04470	Na+/H+ antiporter subunit E		
G7A98_RS04480	Na+/H+ antiporter subunit C		
G7A98_RS01335	Sodium:solute symporter	Sodium and other solute transporters	
G7A98_RS05380	Sodium:solute symporter family protein		
G7A98_RS04245	Sodium:solute symporter family		
G7A98_RS14005	Sodium:solute symporter family protein		
G7A98_RS01475	Sodium:solute symporter family protein		
G7A98_RS05075	Solute:sodium symporter family transporter		
G7A98_RS06695	Sodium:alanine symporter family protein		
G7A98_RS01105	Sodium:bile acid symporter family protein		
G7A98_RS06955	Sodium/calcium antiporter		
G7A98_RS00275	Sodium/proline symporter PutP		
G7A98_RS08890	Sodium:neurotransmitter symporter		
G7A98_RS03100	Sodium:neurotransmitter symporter		
G7A98_RS02010	Sodium:neurotransmitter symporter		
G7A98_RS08345	k+/H+ antiporter	K+/H+ antiporters	
G7A98_RS06095	K+/H+ antiporter		
G7A98_RS04465	K+/H+ antiporter subunit F		
G7A98_RS04475	Multicomponent K+:H+ antiporter subunit D		
G7A98_RS01880	Trk system potassium transporter TrkA	Potassium uptake systems	
G7A98_RS07235	TrkH family potassium uptake protein		
G7A98_RS01885	TrkH family potassium uptake protein		
G7A98_RS08870	TrkH family potassium uptake protein		
G7A98_RS08875	TrkA family potassium uptake protein		
G7A98_RS07615	Potassium channel family protein	Ion channels	
G7A98_RS03215	Chloride channel protein		
G7A98_RS10550	Alanine:cation symporter family protein		
G7A98_RS05870	Cation:proton antiporter		
G7A98_RS00270	Proline dehydrogenase PutA	Proline metabolism	Salt-out strategy
G7A98_RS06195	Glycine betaine/Proline ABC transporter	Glycine Betaine/Proline transport	
G7A98_RS14690	Glycine betaine/Proline transport system substrate-binding protein		
G7A98_RS06190	Glycine betaine/L-proline ABC transporter ATP-binding protein		
G7A98_RS14940	Glycine betaine ABC transporter substrate-binding protein	Glycine betaine uptake	
G7A98_RS06200	Glycine betaine ABC transporter substrate-binding protein		
G7A98_RS08910	BCCT, betaine/carnitine/choline family transporter	Betaine/Carnitine/Choline transport	
G7A98_RS05350	BCCT, betaine/carnitine/choline family transporter		
G7A98_RS01005	BCCT, betaine/carnitine/choline family transporter		
G7A98_RS08520	BCCT, betaine/carnitine/choline family transporter		
G7A98_RS10150	BCCT, betaine/carnitine/choline family transporter		
G7A98_RS14645	BCCT, betaine/carnitine/choline family transporter		
G7A98_RS10005	BCCT, betaine/carnitine/choline family transporter		
G7A98_RS03680	Ectoine synthase	Ectoine biosynthesis	
G7A98_RS06355	Ectoine synthase		
G7A98_RS07620	Ectoine hydroxylase thpD	Ectoine hydroxylation and degradation	
G7A98_RS03705	Ectoine hydroxylase thpD		
G7A98_RS10215	Ectoine hydrolase DoeA		
G7A98_RS14700	Betaine-aldehyde dehydrogenase betB	Betaine biosynthesis	
G7A98_RS10245	Hydroxyectoine utilization dehydratase EutB	Hydroxyectoine metabolism	
G7A98_RS11965	Glutamine synthetase	Glutamine and Glutamate biosynthesis	
G7A98_RS11985	Glutamine synthetase		
G7A98_RS01295	Glutamine synthetase		
G7A98_RS11055	Glutamate synthase large subunit		

The Gene IDs correspond to NCBI RefSeq locus tags. Genes were identified by screening annotations from the IMG and NCBI Prokaryotic Genome Annotation Pipelines and were categorized based on their role in osmotic stress adaptation. These include Na^+/H^+ antiporters, potassium uptake transporters, and compatible solute synthesis and transport systems. Genes were further classified under the salt-in strategy (ion transporters that regulate intracellular concentration of inorganic ions) or the salt-out strategy (compatible solute accumulation systems). BLASTx searches against UniProtKB and Swiss-Prot reference proteomes were performed to validate gene functions.

Table S4. Genes for cross-protection and co-tolerance mechanisms for salinity and heavy metal stress

NCBI Gene ID	Putative Function	Category
G7A98_RS13375	DNA replication/repair protein RecF	DNA repair
G7A98_RS14970	DNA repair protein RecN	
G7A98_RS11090	DNA mismatch repair protein MutS	
G7A98_RS00420	DNA mismatch repair endonuclease MutL	
G7A98_RS03575	DNA repair protein RadA	
G7A98_RS12125	DNA internalization-related competence protein ComEC/Rec2	
G7A98_RS13090	DNA repair protein RecO	
G7A98_RS12320	DNA repair protein RadC	
G7A98_RS09840	DNA repair protein RadC	
G7A98_RS09825	DNA repair protein RadC	
G7A98_RS05020	Biofilm regulation protein phosphatase SiaA	Biofilm Formation
G7A98_RS05015	Biofilm regulation protein kinase SiaB	
G7A98_RS05010	Biofilm regulation phosphoprotein SiaC	
G7A98_RS05005	Biofilm regulation diguanylate cyclase SiaD	
G7A98_RS11415	Catalase	Oxidative stress response
G7A98_RS11085	Ferredoxin	
G7A98_RS10490	Ferredoxin--NADP(+) reductase	
G7A98_RS08940	Glutaredoxin	
G7A98_RS01705	Glutathione peroxidase	
G7A98_RS01090	Glutathione S-transferase	
G7A98_RS08960	Glutathione S-transferase	
G7A98_RS02735	Glutathione S-transferase (Glutaredoxin)	
G7A98_RS05375	Glutathione S-transferase family protein	
G7A98_RS13555	Glutathione synthetase gshB	
G7A98_RS01595	Glutathione transferase	
G7A98_RS11385	Glutathione-dependent disulfide-bond oxidoreductase	
G7A98_RS05355	Glutathione-dependent peroxiredoxin	
G7A98_RS06645	GrxA family glutaredoxin	
G7A98_RS03010	Hydrogen peroxide-inducible genes activator	
G7A98_RS14505	Multicopper oxidase	
G7A98_RS14265	Peroxide stress protein YaaA	
G7A98_RS02335	Peroxiredoxin	
G7A98_RS12385	Alkyl hydroperoxide reductase	
G7A98_RS14330	Thioredoxin	
G7A98_RS07325	Thioredoxin	
G7A98_RS13260	Thioredoxin family protein	
G7A98_RS12390	Thioredoxin reductase trxB	
G7A98_RS12540	Thioredoxin trxA	
G7A98_RS13240	Thioredoxin trxC	
G7A98_RS08850	Thioredoxin-dependent peroxiredoxin	
G7A98_RS00740	Efflux RND transporter periplasmic adaptor subunit	Efflux systems
G7A98_RS00745	Efflux RND transporter permease subunit	
G7A98_RS01560	DHA2 family efflux MFS transporter permease subunit	
G7A98_RS02320	Efflux RND transporter periplasmic adaptor subunit	
G7A98_RS02915	Multidrug efflux MFS transporter	
G7A98_RS03795	DHA2 family efflux MFS transporter permease subunit	
G7A98_RS03970	MATE family efflux transporter MatE	
G7A98_RS04000	PACE efflux transporter	
G7A98_RS05270	Bcr/CflA family efflux MFS transporter	
G7A98_RS05630	MATE family efflux transporter MatE	
G7A98_RS06270	MATE family efflux transporter MatE	
G7A98_RS07160	MATE family efflux transporter MatE	
G7A98_RS07360	Fluoride efflux transporter CrcB	
G7A98_RS07385	Bcr/CflA family multidrug efflux MFS transporter	
G7A98_RS08140	TolC family outer membrane efflux protein	
G7A98_RS08320	Efflux RND transporter permease subunit	
G7A98_RS08325	Efflux RND transporter periplasmic adaptor subunit	
G7A98_RS09265	MATE family efflux transporter MatE	
G7A98_RS09670	TolC family outer membrane efflux protein	
G7A98_RS10155	Efflux RND transporter permease subunit	
G7A98_RS10160	Efflux RND transporter periplasmic adaptor subunit	
G7A98_RS11225	HlyD family efflux transporter periplasmic adaptor subunit	
G7A98_RS14110	HlyD family efflux transporter periplasmic adaptor subunit	
G7A98_RS07130	Universal stress protein	General stress defense
G7A98_RS05060	Universal stress protein	
G7A98_RS05485	Universal stress protein	
G7A98_RS10340	Universal stress protein	
G7A98_RS10625	Universal stress protein	
G7A98_RS00990	Universal stress protein	

The Gene IDs correspond to NCBI RefSeq locus tags. Genes of interest were compiled from literature search and identified by screening IMG and NCBI Prokaryotic Genome Annotation outputs for the bacterium. The genes were further categorized based on function and validated by BLASTx searches against UniProtKB and Swiss-Prot reference proteome database.

Table S5: Qualitative comparison of produced water treatment technologies, including bioremediation by *Modicisalibacter* sp. strain Wilcox

Technology	Target Contaminants	Salinity Tolerance	Heavy Metal Tolerance	Energy Demand	Major Advantages	Key Limitations	References
Bioremediation (*Modicisalibacter* sp. Wilcox - this study)	Hydrocarbons (including BTEX), dissolved metals	Very high (up to 4 M NaCl)	High (multiple metals, mM range)	Low	Environmentally sustainable; simultaneous hydrocarbon degradation and metal removal; can survive across a wide range of conditions	Slower kinetics; sensitive to inhibitors that could be present; requires viable biomass	3, 10, 23
Advanced Oxidation Processes	Organic contaminants (including BTEX)	Low–moderate	Low	High	Rapid contaminant destruction; effective for recalcitrant organics	High energy cost; limited metal tolerance; formation of toxic byproducts	73, 74
Evaporation / evaporation ponds	Volume reduction (salts, metals, organics)	Very high	Very high	Low	Simple operation; widely used for PW volume reduction	Does not destroy contaminants; produces highly concentrated residuals; large land footprint	75, 76
Thermal treatment	Organics	High	High	Very high	Robust; effective across matrices	Extremely energy-intensive; high operational cost	75, 76
Membrane filtration (Reverse Osmosis, Nanofiltration, Ultrafiltration)	Dissolved salts, organics	Moderate	Moderate	High	Produces high-quality effluent	Membrane fouling; poor tolerance to hydrocarbons and metals; pretreatment often required	75, 77, 78
Adsorption (activated carbon, resins)	Organic contaminants (including BTEX), metals	Moderate	Moderate	Low–moderate	Simple operation; rapid removal	Media saturation; requires regeneration; secondary waste	79, 80
Electrochemical treatment	Organic contaminants (including BTEX), metals	Moderate	Moderate	High	High removal efficiency; adaptable operation	Energy-intensive; electrode fouling and scaling	75, 81, 82
